# Continuous flow biocatalysis: synthesis of purine nucleoside esters catalyzed by lipase TL IM from *Thermomyces lanuginosus*†

**DOI:** 10.1039/d4ra00097h

**Published:** 2024-04-04

**Authors:** Shi-Yi Zhang, Guo-Neng Fu, Li-Hua Du, Hang Lin, Ao-Ying Zhang, Han-Jia Xie, Zhi-Kai Sheng, Miao-miao Xue, Bing-lin Yan, Yi Liu, Zhi-Xuan Ruan, Bing-Le Pan, Tong-Yao Zhou, Xi-Ping Luo

**Affiliations:** a College of Pharmaceutical Science, ZheJiang University of Technology Hangzhou 310014 China orgdlh@zjut.edu.cn +86-189-690-693-99; b Zhejiang Provincial Key Laboratory of Chemical Utilization of Forestry Biomass, Zhejiang A&F University Hangzhou 311300 China luoxiping@zafu.edu.cn

## Abstract

Purine nucleoside ester is one of the derivatives of purine nucleoside, which has antiviral and anticancer activities. In this work, a continuous flow synthesis of purine nucleoside esters catalyzed by lipase TL IM from *Thermomyces lanuginosus* was successfully achieved. Various parameters including solvent, reaction temperature, reaction time/flow rate and substrate ratio were investigated. The best yields were obtained with a continuous flow microreactor for 35 min at 50 °C with the substrate ratio of 1 : 5 (nucleosides to vinyl esters) in the solvent of *tert*-amyl alcohol. 12 products were efficiently synthesized with yields of 78–93%. Here we reported for the first time the use of lipase TL IM from *Thermomyces lanuginosus* in the synthesis of purine nucleoside esters. The significant advantages of this methodology are a green solvent and mild conditions, a simple work-up procedure and the highly reusable biocatalyst. This research provides a new technique for rapid synthesis of anticancer and antiviral nucleoside drugs and is helpful for further screening of drug activity.

## Abstract 2

Purine nucleoside ester is one of the derivatives of purine nucleoside, which has antiviral and anticancer activities.

## Graphical abstract

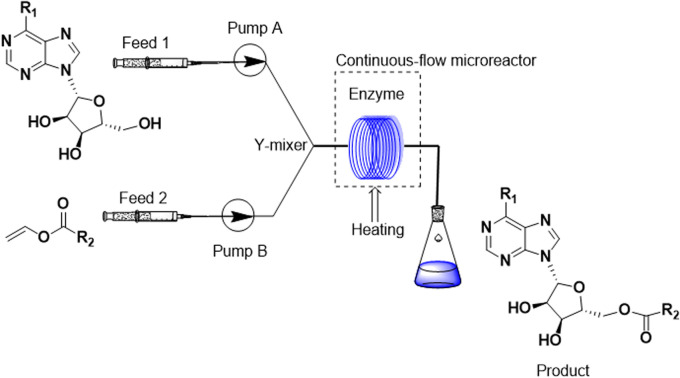

## 1. Introduction

Infections of emerging and reemerging viruses largely and globally affect human health. Therefore, the search for new effective drugs against various viruses with a wide spectrum of activity remains an urgent task.

Purine nucleosides represent an important structural motif in life sciences molecules with remarkable biological properties such as anticancer and antiviral activities.^1,2^ Some of them have been used in clinical therapy, such as vidarabine (antiviral agent), acyclovir (antiviral drug) and cladribine (anticancer drug).^3–5^ Purine nucleosides showed a relatively low permeability and poor uptake into cells.^6–8^ In order to overcome these limitations, it is a common strategy to convert one or more of the hydroxyl (OH) units in purine nucleosides to the corresponding ester groups to prepare purine nucleoside prodrugs for better performance.^9–13^ Valganciclovir, the monoester derivative of ganciclovir, has better efficacy and bioavailability as an antiviral alternative.^14–16^ Purines are mainly involved in metabolic processes, not only used as plant growth regulators, but also have good activity against plant viruses. Purine nucleoside esters can be used as intermediates to synthesize potential drugs that are involved in cell metabolism.^17–19^ The 5′-O-esters of adenosine exhibited antiviral activities against TMV and PVY.^20^ These purine nucleosides have three hydroxyl groups, which makes regional selective acylation more difficult, whereas traditional chemical synthesis requires harsh reaction conditions and complex reaction steps.^21,22^ Accordingly, we hope to develop a highly regioselective method for the synthesis of purine nucleoside esters.

Enzymes as novel natural catalysts have led to a new concept for industrial processes, where biotransformations have substituted a range of traditional synthetic steps generating “greener” routes for the production of pharmaceuticals. Enzymatic approaches are more highly efficient and specific than traditional chemical processes.^23–26^ Many works had been reported on the biocatalysis of purine nucleoside esters. The lipase from *C. antarctica* was used to catalyze 5′-O-acylated adenosines by V. Gotor *et al.*^27^ In this work, the target products were obtained in DMSO for 18–24 h. Zhao *et al.* reported that regioselective acylation of cytarabine catalyzed by *Pseudomonas fluorescens* whole-cells was obtained after 144 h.^28^ Regioselective undecylenoylation of adenosine catalyzed by *Candida antarctica* lipase B needed 23 h.^29^ These works are of great significance for the biocatalysis of purine nucleoside esters. As we can see, biocatalytic method of purine nucleoside esters sometimes required long reaction times or toxic solvents. So whether a more efficient and green method to synthesize purine nucleoside esters can be found has caught our attention.

**Scheme 1:**
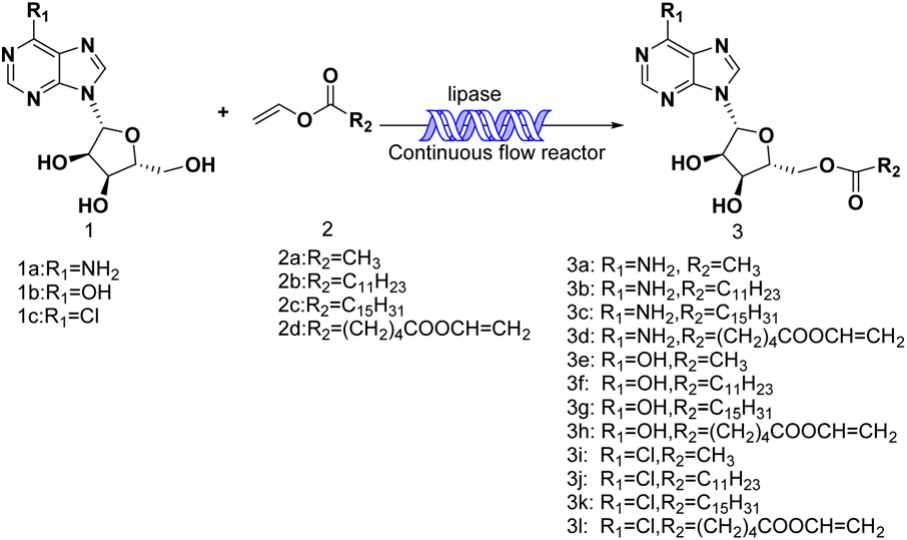
Synthesis and design of purine nucleoside esters catalyzed by lipase TL IM in continuous-flow microreactors.

Continuous flow technology, as one of the top 10 sustainable development technologies for the future, has gradually matured in its industrial applications. From the perspective of green chemistry, continuous flow technology has been proved to be beneficial for process optimization, offering new synthesis routes, enhancing reaction selectivity, reducing downstream processing costs, and improving process safety.^30,31^ The encounter of continuous flow technology and biocatalysis has endowed green catalytic synthesis with greater sustainable value. Enzymatic technique integrates with continuous-flow technology, which enhances the reaction process, decrease reaction times and increase the productivity.^32–35^ Could the integration of enzymatic method and continuous-flow technology effectively promote the synthesis of purine nucleoside esters? In our previous studies, some works about continuous-flow enzymatic methods were reported.^35–38^ The objective of this research was to find an efficient and green continuous-flow enzymatic method of purine nucleoside esters (Scheme 1).

## 2. Results and discussion

### 2.1. Effect of reaction media

Firstly, we explored the enzymatic synthesis of purine nucleoside esters in continuous flow reactors. Taking the reaction of adenosine and vinyl laurate as a model reaction, the reaction conditions were discussed. Both the catalytic activity of enzyme and the reaction rate are affected by the reaction medium. In order to find the best reaction medium, we had tried many solvents such as *tert*-amyl alcohol, methanol, ethanol, isopropyl alcohol, cyclohexane, *n*-hexane, acetonitrile and DMSO. After experiments, we found that the reaction effects of *tert*-amyl alcohol, isopropyl alcohol, acetonitrile and DMSO were better, among which *tert*-amyl alcohol had the best reaction result (Table 1). Purine nucleosides did not dissolve in these solvents, such as *n*-hexane, cyclohexane, methanol and ethanol. Enzymes also had the problem of clumping in the above solvents. These factors resulted in the yields of 0. Therefore, we synthesized purine nucleoside esters catalyzed by lipase TL IM in *tert*-amyl alcohol.

**Table 1:** Effect of reaction solvent on the synthesis of purine nucleoside esters in continuous-flow microreactors^a^

Entry	Solvent	Catalysts	Log *P*	Yield^b^ (%)
1	*tert*-Amyl alcohol	None	1.04	n.d.
2	*tert*-Amyl alcohol	Lipase TL IM	1.04	68.4 ± 0.9
3	DMSO	Lipase TL IM	−1.3	49.7 ± 1.4
4	Acetonitrile	Lipase TL IM	−0.33	47.1 ± 1.7
5	Isopropyl alcohol	Lipase TL IM	0.28	21.7 ± 1.1
6	*n*-Hexane	Lipase TL IM	3.94	n.d.
7	Cyclohexane	Lipase TL IM	3.44	n.d.
8	Methanol	Lipase TL IM	−0.76	n.d.
9	Ethanol	Lipase TL IM	−0.24	n.d.

a Reaction conditions: continuous-flow reactor, feed 1, 10 mL solvent contained 5.0 mmol adenosine (1a); feed 2, 10 mL solvent contained 35.0 mmol vinyl laurate (2b), enzyme 0.870 g (catalyst reactivity: 250 IUN·g^−1^), flow rate 15.6 μL min^−1^, residence time 40 min, 50 °C.

b Isolated yield. Yield: 100 × (actual received quantity/ideal calculated quantity). The data are presented as average ± SD of triplicate experiments.

### 2.2. Effect of substrate ratio

**Fig. 1:**
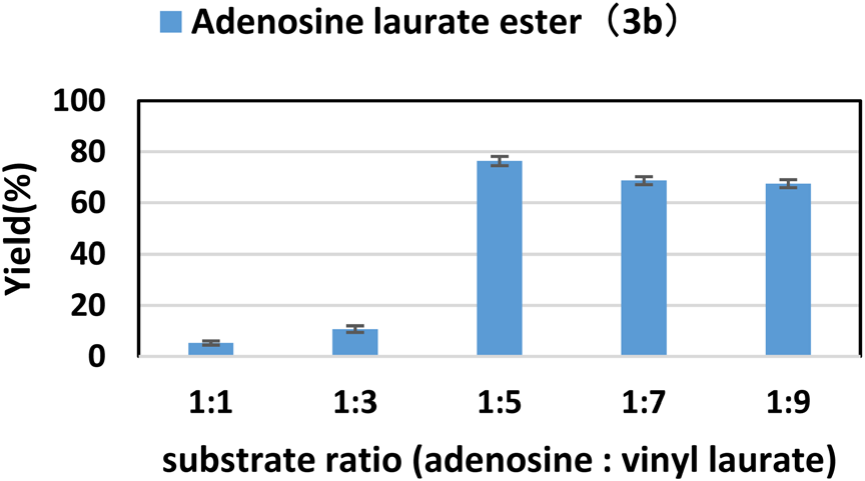
The influence of substrate ratio (adenosine: vinyl laurate) on the synthesis of adenosine laurate ester catalyzed by lipase TL IM in continuous-flow microreactors.

In the enzymatic reaction, the substrate ratio has a great influence on the catalytic efficiency of the enzyme and the product yields. In this study, the concentration of adenosine was determined, and subsequently, the concentration of vinyl laurate was systematically adjusted to yield a range of substrate ratios (adenosine: vinyl laurate) spanning from 1 : 1 to 1 : 9. It could be seen from Fig. 1 that as the ratio of vinyl laurate increased, the yield of the target product gradually increased. The maximum yield of 76.4% was obtained when the molar ratio (adenosine: vinyl laurate) was 1 : 5. However, when the molar ratio continued to increase, the yield of the product had even slightly decreased. Since the glycosyl of adenosine has multiple hydroxyl groups, as the substrate ratio continued to increase, more by-products would be produced. Therefore, in the further condition exploration, we chose adenosine : vinyl laurate = 1 : 5 as the optimal substrate ratio.

### 2.3. Effect of reaction temperature

**Fig. 2:**
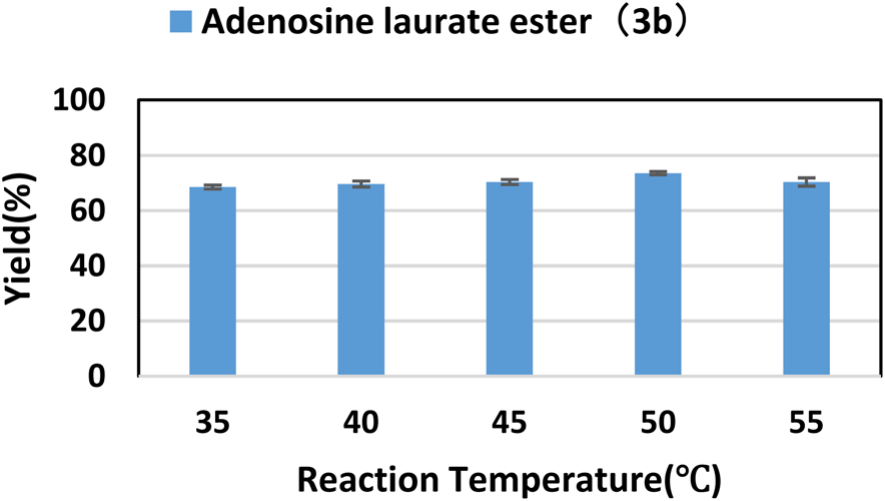
The influence of reaction temperature on the synthesis of adenosine laurate ester catalyzed by lipase TL IM in continuous-flow microreactors.

Enzyme activity has an optimal temperature. Temperature that is too high or too low would reduce enzyme activity; in addition, temperature that is too high can also cause enzyme inactivation or even denaturation. At the same time, with the increase of temperature, the viscosity of the system decreases, the diffusion rate increases, and the rate of chemical reaction will also accelerate. Using lipase TL IM as catalyst, flow reaction was carried out in *tert*-amyl alcohol at reaction temperatures of 35 °C to 55 °C for 40 min. As shown in the Fig. 2, when the reaction temperature was 50 °C, the best yield was obtained.

### 2.4. Effect of residence time

**Fig. 3:**
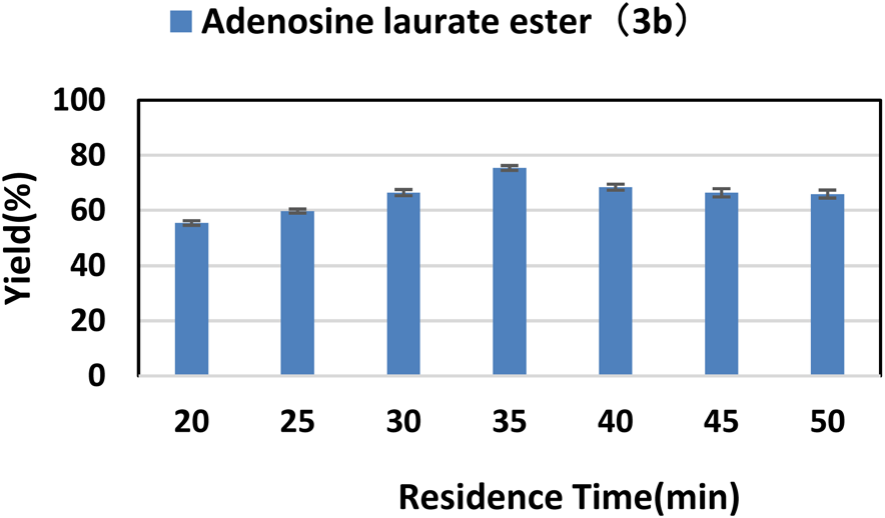
The influence of residence time on the synthesis of adenosine laurate ester catalyzed by lipase TL IM in continuous-flow microreactors.

Residence time is an important parameter in microfluidic reaction. The catalysis of the enzyme on the reaction requires a sufficiently long contact time with the substrate, but too long a time may result in the formation of by-products. The effect of residence time on enzymatic synthesis of purine nucleoside esters was studied by increasing the residence time from 20 min to 50 min. As we can see from Fig. 3, the yield was the highest after 35 min; as the reaction time continued to increase, the content of main products gradually reduced. We guessed that the resulting by-products might be 3′-O-esters of purine nucleosides, 2′-O-esters of purine nucleosides and 2′,3′,5′-O-esters of purine nucleosides. The increase of time leaded to the generation of by-products.

### 2.5. The effect of enzyme reusability on the reaction

**Fig. 4:**
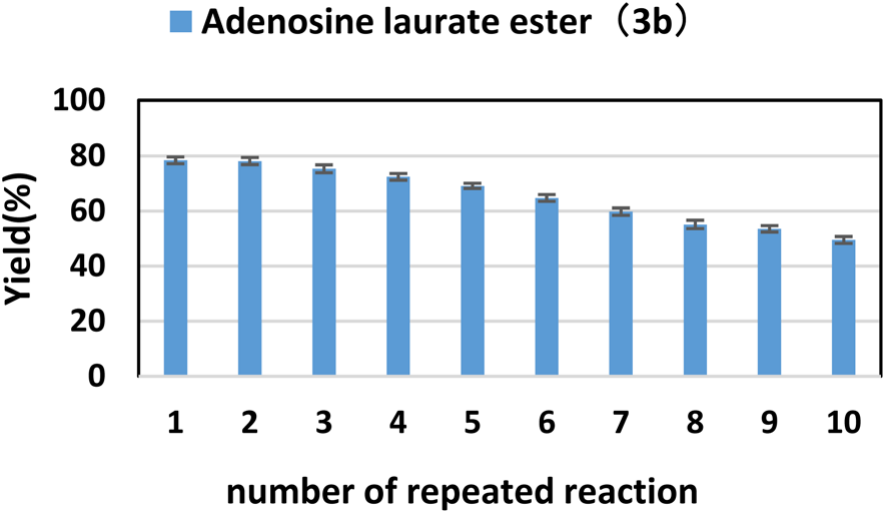
The influence of number of repeated reaction on the synthesis of adenosine laurate ester catalyzed by lipase TL IM in continuous-flow microreactors.

Because enzymes can be recycled to reduce production costs, enzyme reusability is an important aspect of enzyme reactions. Under optimal reaction conditions, we investigated the yield of adenosine laurate ester (3b) over 10 reaction cycles to determine the repeatability of lipase TL IM. As shown in Fig. 4, with the increase of the number of cycles, the catalytic activity of lipase TL IM gradually decreased, and the yield of the product was still 49.5% at the last cycle. This shows that lipase TL IM has good repeatability.

### 2.6. The effect of substrate structure on the reaction

**Fig. 5:**
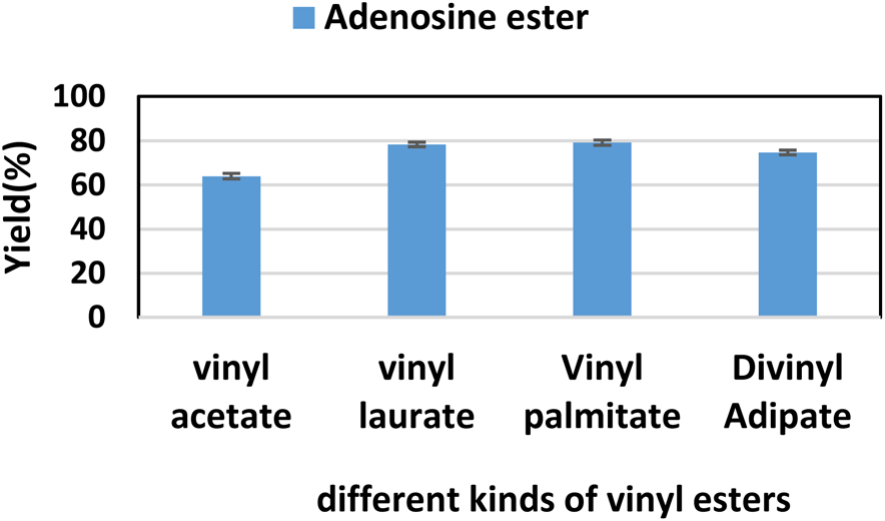
The effect of different kinds of vinyl esters on the synthesis of adenosine laurate ester catalyzed by lipase TL IM in continuous-flow microreactors.

**Fig. 6:**
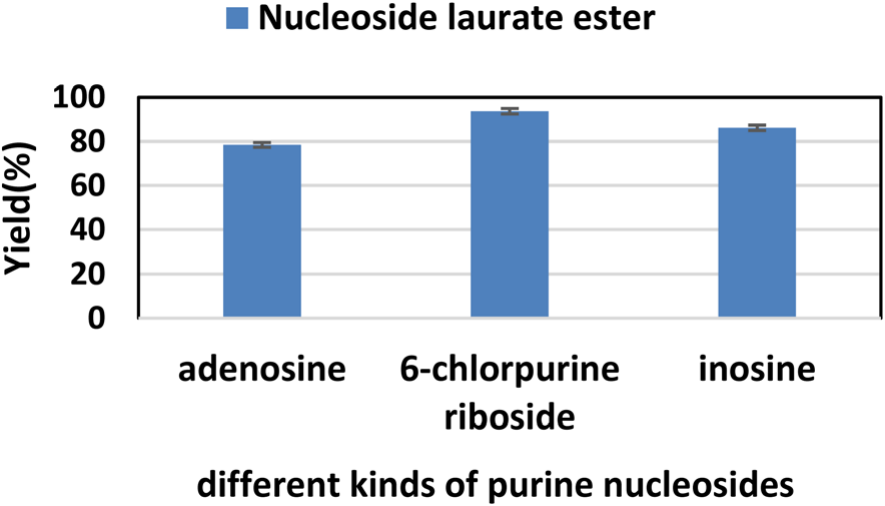
The effect of different kinds of purine nucleosides on the synthesis of adenosine laurate ester catalyzed by lipase TL IM in continuous-flow microreactors.

Firstly, we studied the effect of receptor structure on the synthesis of purine nucleoside esters (Fig. 5). Choosing adenosine as the donor, we found that the longer the carboxyl chain of vinyl ester, the higher the yield of the target product. The yield from adenosine to vinyl palmitate (79.2%) was almost equal to vinyl laurate (78.4%). Meanwhile, the yield of adenosine to vinyl acetate with the shortest chain length was only 64%. Then, using vinyl laurate as acceptor, we continued to study the effect of different substituents on purine nucleosides (Fig. 6). We found that under the same reaction conditions, the yield of adenosine was the lowest (78.4%). This is due to the presence of a primary amino group on the adenosine, which leads to the formation of some additional by-products. The reaction yield of 6-chlorpurine riboside with vinyl laurate was the highest (93.7%), and almost no byproducts were produced.

### 2.7. Comparing the synthesis of purine nucleoside esters from purine nucleosides and vinyl esters in a continuous-flow reaction and a batch reaction

To investigate the effect of enzymatic reactions in different reactors, we optimized the conditions of enzymatic reactions in a continuous-flow reaction and a batch reaction (Table 2). In the batch reaction (method A), it took about 26 h to obtain the desired yields. However, in the continuous-flow reaction (method B), better yields can be obtained in 35 minutes. Space time yield (STY) is a common index to evaluate the production effect of different reactors. Therefore, the reaction of adenosine with vinyl laurate was used as a model reaction to calculate STY (g L^−1^ h^−1^) to evaluate the productivity of two reactors. It was found that STY of the continuous-flow reactor was higher. The results showed that the continuous-flow reactor was more beneficial to improve the efficiency of enzymatic synthesis of purine nucleoside esters.STY = *m*_p_ × *T*^−1^ × *V*_R_^−1^STY(Method A) = 1.125 g L^−1^ h^−1^STY(Method B) = 62.06 g L^−1^ h^−1^where *m*_p_ is the mass of the generated product (g), *T* is the residence time (h), and *V*_R_ is the reactor volume (L).

**Table 2:** Batch and continuous-flow synthesis of purine nucleoside esters catalyzed by lipase TL IM

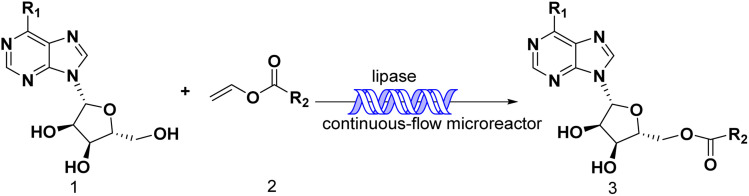					
Entry	R_1_	R_2_	Method^a^	Time	Yield^b^ (%)
1	NH_2_	CH_3_	A	26 h	59.1 ± 0.7(3a)
			B	35 min	64.0 ± 1.1(3a)
2	NH_2_	C_11_H_23_	A	26 h	64.5 ± 0.6(3b)
			B	35 min	78.4 ± 0.9(3b)
3	NH_2_	C_15_H_31_	A	26 h	63.1 ± 0.4(3c)
			B	35 min	79.2 ± 1.6(3c)
4	NH_2_	(CH_2_)_4_COOCH=CH_2_	A	26 h	60.6 ± 0.5(3d)
			B	35 min	74.7 ± 0.7(3d)
5	OH	CH_3_	A	26 h	58.3 ± 0.9(3e)
			B	35 min	67.5 ± 0.6(3e)
6	OH	C_11_H_23_	A	26 h	77.4 ± 0.7(3f)
			B	35 min	86.1 ± 1.2(3f)
7	OH	C_15_H_31_	A	26 h	78.6 ± 1.3(3g)
			B	35 min	88.7 ± 0.9(3g)
8	OH	(CH_2_)_4_COOCH=CH_2_	A	26 h	73.2 ± 0.7(3h)
			B	35 min	77.3 ± 1.4(3h)
9	Cl	CH_3_	A	26 h	61.7 ± 0.7(3i)
			B	35 min	71.4 ± 0.8(3i)
10	Cl	C_11_H_23_	A	26 h	85.3 ± 1.3(3j)
			B	35 min	93.7 ± 0.9(3j)
11	Cl	C_15_H_31_	A	26 h	84.6 ± 0.8(3k)
			B	35 min	92.9 ± 1.1(3k)
12	Cl	(CH_2_)_4_COOCH=CH_2_	A	26 h	72.3 ± 0.8(3l)
			B	35 min	80.5 ± 1.6(3l)

a Method A: batch reactor, 5.0 mmol purine nucleoside (1) and 25.0 mmol vinyl esters (2) added to 20 mL *tert*-amyl alcohol in a 50 mL erlenmeyer flask, 0.870 g lipase TL IM M (catalyst reactivity: 250 IUN g^−1^), 26 h, 50 °C. Method B: continuous-flow reactor, feed 1, 10 mL *tert*-amyl alcohol contained 5.0 mmol purine nucleoside (1); feed 2, 10 mL *tert*-amyl alcohol contained 25.0 mmol vinyl esters (2), lipase TL IM 0.870 g (catalyst reactivity: 250 IUN g^−1^), flow rate 18.3 μL min^−1^, residence time 35 min, 50 °C.

b Isolated yield. Yield: 100 × (actual received quantity/ideal calculated quantity). The data are presented as average ± SD of triplicate experiments.

### 2.8. The toxicologically evaluation

We got the toxicological data of purine nucleosides from the Chemical Toxicity Database and the Toxnet. Adenosine might cause the consequences of arrhythmia, headache, bronchiolar constriction and rapid heartbeat. In addition, adenosine might cause drug-induced liver damage. Inosine might cause stomach upset and diarrhoea. The oral LD_50_ of adenosine in the rat was greater than 20 mg kg^−1^. The dose of inosine's oral LD_50_ test in the rat was greater than 10 mg kg^−1^. The intraperitoneal LD_50_ of 6-chloropurine riboside in the rat was greater than 800 mg kg^−1^.

We used the analysis function of the QSAR Toolbox to predict the toxicity of the impurities. The structures of 3′-O-esters of purine nucleosides and 2′-O-esters of purine nucleosides were similar to puromycin (hepatotoxicity) and puromycin aminonucleoside (renal toxicity). 2′,3′,5′-O-esters of purine nucleosides were similar to auranofin (renal toxicity). The dose of puromycin's intraperitoneal LD_50_ test in the mouse was 500 mg kg^−1^. The dose of puromycin aminonucleoside's intraperitoneal DNA inhibition test in the mouse was 83 mg kg^−1^. The dose of auranofin's oral LD_50_ test in the rat was 265 mg kg^−1^.

### 2.9. The nitrosamine risk assessment

We conducted a nitrosamine risk assessment from two aspects.

The risk assessment of process materials was the first step. No sodium nitrite or other nitrite reagents were used in the whole reaction process. The preparation process of purine nucleosides, vinyl esters and *tert*-amyl alcohol did not use amine compounds and nitroso compounds. The risk of nitrosamine impurity contamination was low.

The second step was the risk assessment of the reaction process. *tert*-Amyl alcohol was a neutral solvent. No nitroso compounds were used in this reaction, only purine nucleosides were used. And it was not a strong acidic reaction. It was assumed that nitrosamine impurities were formed by the reactions of purine nucleosides and very small amount of nitroso in the water under the acidic condition. The content of nitrosamine impurities would be very low and it could be considered to test the finished product for further confirmation.

## 3. Materials and methods

### 3.1. Materials

All chemicals in this study were obtained from commercial sources and did not require further purification. Lipase TL IM form *Thermomyces lanuginosus* was purchased from Novo Nordisk. Adenosine was purchased from Macklin (Shanghai, China), inosine was purchased from Accela (Shanghai, China), 6-chloropurine nucleoside was purchased from Aladdin (Shanghai, China). Vinyl acetate was purchased from SCRC (Shanghai, China), vinyl laurate from Aladdin (Shanghai, China), vinyl palmitate and divinyl adipate from TCI (Tokyo, Japan). Harvard Instrument PHD 2000 syringe pump was purchased from Harvard University (Holliston, MA, USA). The flow reactor and Y-mixer were purchased from Beijing Haigui Medical Engineering Design Co., Ltd. (Beijing China). A 400 MHz NMR spectrometer (Billerica, MA, USA) were also used in this study.

### 3.2. Experimental setup and experiment conditions

**Fig. 7:**
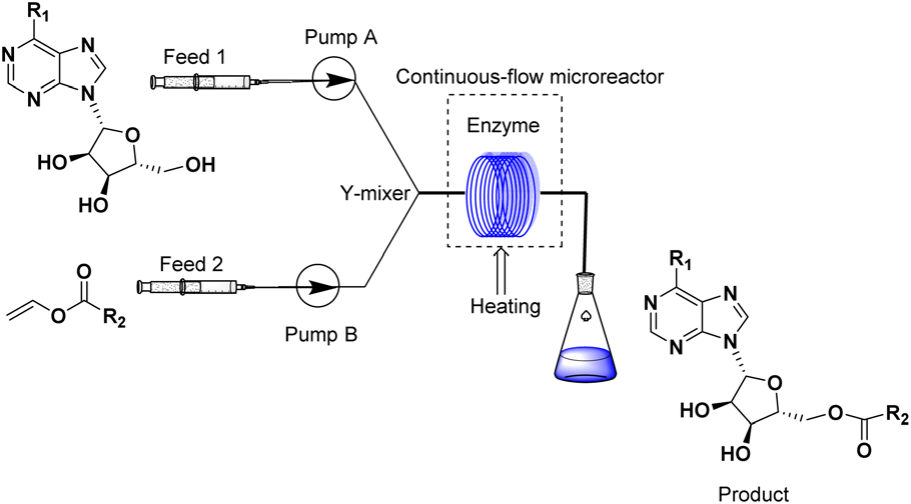
Equipment for the enzymatic synthesis of purine nucleoside esters in continuous-flow microreactors.

The apparatus configuration used for the synthesis of purine nucleoside esters in a continuous flow microreactor is shown in Fig. 7. The experimental Apparatus consisted of five main components: a syringe pump (Harvard Apparatus Dr. 2000), substrate injectors, a Y-mixer, a flow reactor, and a product collector. Preparation: the flow reactor with an inner diameter of 2 mm was first filled with 0.870 g lipase TL IM, particle size 0.3–1.0 mm, reactivity 250 IUN g ^−1^, and then immersed in a constant temperature water bath at 50 °C. Work began: 5.0 mmol purine riboside dissolved in 10 mL *tert*-amyl alcohol in feed 1 and 25.0 mmol vinyl esters dissolved in 10 mL *tert*-amyl alcohol in feed 2. The two solutions were intersected in a Y-type mixer, and the mixed flow was passed through the flow reactor at the flow rate of 18.3 μL min^−1^ with a residence time of 35 min. Finally, the reaction solution was collected and dried by evaporation. The products were separated by silica gel chromatography (eluent: dichloromethane/methanol from 30/1 to 24/1). The main products were determined by ^1^H NMR and ^13^C NMR.

### 3.3. Analytical methods

#### 3.3.1. Thin-layer chromatography (TLC)

TLC analysis with dichloromethane/methanol 10/1 (v/v) as the eluent. The results were detected by UV irradiation at 254 nm.

#### 3.3.2. Nuclear magnetic resonance (NMR)

The product obtained by column chromatography separation and purification was subjected to ^1^H NMR and ^13^C NMR structure confirmation on NMR spectrometer.

##### 3.3.2.1 (5-(6-Amino-9*H*-purin-9-yl)-3,4-dihydroxytetrahydrofuran-2-yl)methyl acetate (3a)

White solid, ^1^H NMR (400 MHz, DMSO-*d*_6_) *δ* 8.32 (s, 1H), 8.16 (d, *J* = 3.6 Hz, 1H), 7.33 (s, 2H), 5.93 (s, 1H), 5.61 (s, 1H), 5.43–5.35 (m, 1H), 4.69 (s, 1H), 4.18 (d, *J* = 80.4 Hz, 4H), 2.01 (s, 3H); ^13^C NMR (101 MHz, DMSO) *δ* 170.68, 156.54, 153.14, 149.80, 140.20, 119.60, 88.19, 81.94, 73.29, 70.75, 64.37, 21.05.

##### 3.3.2.2 (5-(6-Amino-9*H*-purin-9-yl)-3,4-dihydroxytetrahydrofuran-2-yl)methyl dodecanoate (3b)

White solid, ^1^H NMR (400 MHz, DMSO-*d*_6_) *δ* 8.30 (s, 1H), 8.14 (s, 1H), 7.30 (s, 2H), 5.90 (d, *J* = 4.9 Hz, 1H), 5.59 (d, *J* = 5.6 Hz, 1H), 5.38 (d, *J* = 5.4 Hz, 1H), 4.66 (q, *J* = 5.2 Hz, 1H), 4.33 (dd, *J* = 11.9, 3.7 Hz, 1H), 4.29–4.23 (m, 1H), 4.19 (dd, *J* = 11.9, 6.1 Hz, 1H), 4.11–4.03 (m, 1H), 2.32–2.24 (m, 2H), 1.51–1.43 (m, 2H), 1.21 (d, *J* = 5.8 Hz, 16H), 0.85 (t, *J* = 6.7 Hz, 3H); ^13^C NMR (101 MHz, DMSO) *δ* 173.26, 156.54, 153.11, 149.93, 140.14, 119.59, 88.22, 81.92, 73.34, 70.70, 64.14, 33.77, 31.75, 29.43, 29.31, 29.16, 29.11, 28.84, 24.83, 22.56, 14.43.

##### 3.3.2.3 (5-(6-Amino-9*H*-purin-9-yl)-3,4-dihydroxytetrahydrofuran-2-yl)methyl palmitate (3c)

White solid, ^1^H NMR (400 MHz, DMSO-*d*_6_) *δ* 8.31 (s, 1H), 8.14 (s, 1H), 7.33 (s, 2H), 5.91 (d, *J* = 4.8 Hz, 1H), 5.59 (d, *J* = 5.8 Hz, 1H), 5.38 (d, *J* = 5.5 Hz, 1H), 4.66 (q, *J* = 5.2 Hz, 1H), 4.37–4.14 (m, 3H), 4.07 (q, *J* = 5.2, 4.8 Hz, 1H), 2.28 (t, *J* = 7.4 Hz, 2H), 1.47 (p, *J* = 6.7 Hz, 2H), 1.29–1.15 (m, 24H), 0.85 (t, *J* = 6.6 Hz, 3H); ^13^C NMR (101 MHz, DMSO) *δ* 173.22, 156.54, 153.07, 149.77, 140.13, 119.62, 88.23, 81.91, 73.35, 70.73, 64.18, 33.77, 31.77, 29.52, 29.49, 29.44, 29.34, 29.19, 29.15, 28.87, 24.84, 22.57, 14.42.

##### 3.3.2.4 (5-(6-Amino-9*H*-purin-9-yl)-3,4-dihydroxytetrahydrofuran-2-yl)methyl vinyl adipate (3d)

Yellowish solid, ^1^H NMR (400 MHz, DMSO-*d*_6_) *δ* 8.32 (s, 1H), 8.16 (s, 1H), 7.32 (s, 2H), 7.21 (t, *J* = 10.2 Hz, 1H), 5.92 (m, 1H), 4.89 (s, 1H), 4.67 (s, 2H), 4.38–4.31 (m, 1H), 4.29 (s, 1H), 4.22 (s, 1H), 4.07 (s, 1H), 2.86 (s, 1H), 2.42 (s, 1H), 2.28 (s, 3H), 2.17 (s, 1H), 1.16 (d, *J* = 49.4 Hz, 4H); ^13^C NMR (101 MHz, DMSO) *δ* 173.68, 170.75, 156.53, 153.10, 149.78, 141.65, 140.17, 119.60, 98.35, 88.28, 81.93, 73.39, 70.74, 64.24, 34.09, 33.46, 24.46, 24.22.

##### 3.3.2.5 (3,4-Dihydroxy-5-(6-hydroxy-9*H*-purin-9-yl)tetrahydrofuran-2-yl)methyl acetate (3e)

White solid, ^1^H NMR (400 MHz, DMSO-*d*_6_) *δ* 12.43 (s, 1H), 8.29 (s, 1H), 8.09 (s, 1H), 5.91 (d, *J* = 5.0 Hz, 1H), 5.65 (s, 1H), 4.58 (t, *J* = 5.1 Hz, 1H), 4.31 (dd, *J* = 11.9, 3.6 Hz, 1H), 4.19 (dq, *J* = 11.8, 6.0, 5.5 Hz, 2H), 4.10 (dt, *J* = 5.8, 3.9 Hz, 1H), 3.17 (s, 1H), 2.02 (s, 3H); ^13^C NMR (101 MHz, DMSO-*d*_6_) *δ* 170.70, 157.06, 148.70, 146.43, 139.34, 124.95, 88.15, 82.17, 73.75, 70.70, 64.30, 21.05.

##### 3.3.2.6 (3,4-Dihydroxy-5-(6-hydroxy-9*H*-purin-9-yl)tetrahydrofuran-2-yl)methyl dodecanoate (3f)

White solid, ^1^H NMR (400 MHz, DMSO-*d*_6_) *δ* 12.42 (s, 1H), 8.27 (s, 1H), 8.07 (s, 1H), 5.89 (d, *J* = 4.9 Hz, 1H), 5.63 (d, *J* = 5.8 Hz, 1H), 5.43–5.38 (m, 1H), 4.55 (q, *J* = 4.3 Hz, 1H), 4.31 (dd, *J* = 12.0, 3.7 Hz, 1H), 4.19 (q, *J* = 6.1 Hz, 2H), 4.09 (q, *J* = 4.8 Hz, 1H), 2.29 (t, *J* = 7.4 Hz, 2H), 1.48 (p, *J* = 6.9 Hz, 2H), 1.30–1.18 (m, 16H), 0.85 (s, 2H), 0.84 (d, *J* = 13.4 Hz, 1H). ^13^C NMR (101 MHz, DMSO) *δ* 173.23, 157.02, 148.66, 146.37, 139.23, 124.97, 88.17, 82.12, 73.83, 70.67, 64.12, 33.78, 31.76, 29.45, 29.34, 29.18, 29.15, 28.86, 24.86, 22.57, 14.42.

##### 3.3.2.7 (3,4-Dihydroxy-5-(6-hydroxy-9*H*-purin-9-yl)tetrahydrofuran-2-yl)methyl palmitate (3g)

White solid. ^1^H NMR (400 MHz, DMSO-*d*_6_) *δ* 8.26 (s, 1H), 8.06 (s, 1H), 5.88 (d, *J* = 4.9 Hz, 1H), 5.65–5.60 (m, 1H), 5.40 (s, 1H), 4.54 (t, *J* = 5.0 Hz, 1H), 4.30 (dd, *J* = 11.9, 3.7 Hz, 1H), 4.23–4.14 (m, 2H), 4.12–4.04 (m, 1H), 2.50 (t, *J* = 2.0 Hz, 1H), 2.29 (t, *J* = 7.3 Hz, 2H), 1.47 (q, *J* = 7.1 Hz, 2H), 1.35–1.18 (m, 24H), 0.84 (t, *J* = 6.7 Hz, 3H). ^13^C NMR (101 MHz, chloroform-*d*) *δ* 172.78, 156.58, 148.22, 145.93, 138.79, 124.53, 87.74, 81.68, 73.39, 70.23, 63.69, 33.34, 31.33, 29.08, 29.05, 29.01, 28.91, 28.75, 28.72, 28.60, 28.44, 24.42, 22.13, 13.97.

##### 3.3.2.8 (3,4-Dihydroxy-5-(6-hydroxy-9*H*-purin-9-yl)tetrahydrofuran-2-yl)methyl vinyl adipate (3h)

White solid, ^1^H NMR (400 MHz, DMSO-*d*_6_) *δ* 12.42 (s, 1H), 8.28 (s, 1H), 8.09 (s, 1H), 7.20 (dd, *J* = 14.0, 6.3 Hz, 1H), 5.91 (d, *J* = 4.8 Hz, 1H), 5.84–5.14 (m, 1H), 4.89 (d, *J* = 1.6 Hz, 1H), 4.85 (d, *J* = 1.6 Hz, 1H), 4.66–4.54 (m, 2H), 4.32 (dd, *J* = 11.9, 3.7 Hz, 1H), 4.25–4.16 (m, 2H), 4.11 (dt, *J* = 5.8, 4.0 Hz, 1H), 2.46–2.38 (m, 2H), 2.38–2.30 (m, 2H), 1.55 (tt, *J* = 7.2, 3.7 Hz, 4H); ^13^C NMR (101 MHz, DMSO-*d*_6_) *δ* 173.03, 170.69, 157.07, 148.68, 146.41, 141.65, 139.30, 124.96, 98.47, 88.19, 82.14, 73.79, 70.68, 64.20, 33.35, 33.09, 24.11, 23.86.

##### 3.3.2.9 (5-(6-Chloro-9*H*-purin-9-yl)-3,4-dihydroxytetrahydrofuran-2-yl)methyl acetate (3i)

White solid, ^1^H NMR (400 MHz, DMSO-*d*_6_) *δ* 8.87 (s, 1H), 8.81 (s, 1H), 6.07 (d, *J* = 4.6 Hz, 1H), 5.71 (d, *J* = 5.7 Hz, 1H), 5.48 (d, *J* = 5.5 Hz, 1H), 4.71 (q, *J* = 5.1 Hz, 1H), 4.38–4.12 (m, 4H), 2.01 (s, 3H); ^13^C NMR (101 MHz, DMSO) *δ* 170.68, 152.24, 151.95, 149.88, 146.49, 131.92, 88.99, 82.35, 73.54, 70.62, 64.17, 21.02.

##### 3.3.2.10 (5-(6-Chloro-9*H*-purin-9-yl)-3,4-dihydroxytetrahydrofuran-2-yl)methyl dodecanoate (3j)

White solid, ^1^H NMR (400 MHz, DMSO-*d*_6_) *δ* 8.86 (s, 1H), 8.80 (s, 1H), 6.07 (d, *J* = 4.5 Hz, 1H), 5.69 (d, *J* = 5.4 Hz, 1H), 5.45 (d, *J* = 5.6 Hz, 1H), 4.71 (q, *J* = 5.0 Hz, 1H), 4.32 (qd, *J* = 11.5, 11.1, 4.7 Hz, 2H), 4.26–4.20 (m, 1H), 4.16 (q, *J* = 4.9 Hz, 1H), 2.25 (td, *J* = 7.4, 2.4 Hz, 2H), 1.44 (p, *J* = 6.8 Hz, 2H), 1.30–1.15 (m, 16H), 0.81 (t, *J* = 6.7 Hz, 3H); ^13^C NMR (101 MHz, DMSO) *δ* 173.12, 152.16, 151.93, 149.90, 146.46, 131.94, 89.07, 82.30, 73.62, 70.58, 63.90, 33.76, 31.75, 29.44, 29.33, 29.17, 29.13, 28.86, 24.82, 22.55, 14.31.

##### 3.3.2.11 (5-(6-Chloro-9*H*-purin-9-yl)-3,4-dihydroxytetrahydrofuran-2-yl)methyl palmitate (3k)

White solid, ^1^H NMR (400 MHz, DMSO-*d*_6_) *δ* 8.87 (s, 1H), 8.82 (s, 1H), 6.06 (d, *J* = 4.5 Hz, 1H), 5.68 (d, *J* = 5.4 Hz, 1H), 5.44 (d, *J* = 5.6 Hz, 1H), 4.71 (q, *J* = 5.0 Hz, 1H), 4.38–4.19 (m, 3H), 4.14 (td, *J* = 5.6, 3.6 Hz, 1H), 3.33 (d, *J* = 1.8 Hz, 4H), 2.25 (td, *J* = 7.4, 2.8 Hz, 2H), 1.44 (p, *J* = 7.1 Hz, 2H), 1.22 (d, *J* = 3.5 Hz, 13H), 1.17 (s, 7H), 0.89–0.81 (m, 3H); ^13^C NMR (101 MHz, DMSO) *δ* 173.19, 152.24, 151.98, 149.88, 146.55, 131.94, 89.03, 82.32, 73.55, 70.56, 63.89, 33.76, 31.76, 29.51, 29.49, 29.45, 29.41, 29.30, 29.17, 29.09, 28.82, 24.83, 22.56, 14.40.

##### 3.3.2.12 (5-(6-Chloro-9*H*-purin-9-yl)-3,4-dihydroxytetrahydrofuran-2-yl)methyl vinyl adipate (3l)

Yellow solid, ^1^H NMR (400 MHz, DMSO-*d*_6_) *δ* 8.81 (d, *J* = 22.4 Hz, 2H), 7.16 (dd, *J* = 14.0, 6.3 Hz, 1H), 6.08 (d, *J* = 4.4 Hz, 1H), 5.71 (d, *J* = 5.5 Hz, 1H), 5.47 (d, *J* = 5.6 Hz, 1H), 4.82 (dd, *J* = 14.0, 1.6 Hz, 1H), 4.73 (q, *J* = 5.0 Hz, 1H), 4.58 (dd, *J* = 6.4, 1.6 Hz, 1H), 4.32 (dtd, *J* = 29.1, 12.0, 4.7 Hz, 3H), 4.18 (td, *J* = 5.4, 3.5 Hz, 1H), 2.39 (t, *J* = 6.4 Hz, 2H), 2.31 (t, *J* = 5.3 Hz, 2H), 1.58–1.48 (m, 4H); ^13^C NMR (101 MHz, DMSO) *δ* 172.96, 170.57, 152.13, 151.89, 149.90, 146.36, 141.55, 131.92, 98.24, 89.07, 82.29, 73.64, 70.60, 64.01, 33.35, 33.08, 24.08, 23.82.

## 4. Conclusions

Purine nucleosides represent an important structural motif in life science molecules with remarkable biological properties. One of the derivatives of this compound is purine nucleoside ester, which has many potential effects such as anticancer and antiviral activities. Some of them have been used in clinical therapy as antiviral and anticancer drugs. In this work, the continuous flow synthesis of purine nucleoside esters catalyzed by lipase TL IM from *Thermomyces lanuginosus* was successfully achieved. Various parameters including solvent, reaction temperature, reaction time/flow rate and substrate ratio were investigated. The best yields of purine nucleoside esters (78–93%) were obtained for 35 min with the substrate ratio of 1 : 5 (nucleosides to vinyl esters) at 50 °C in the solvent of *tert*-amyl alcohol. 3 purine nucleosides (adenosine, inosine, 6-chloropurine) and 4 vinyl esters (vinyl acetate, vinyl laurate, vinyl palmitate, divinyl adipate) were used to synthesize 12 purine nucleoside esters. Here we reported for the first-time the use of lipase TL IM from *Thermomyces lanuginosus* in the synthesis of purine nucleoside esters. The significant advantages of this methodology are green solvent (*tert*-amyl alcohol) and mild conditions (50 °C), easy handling of the highly reusable biocatalyst and environmental-friendly processes. The encounter of continuous flow technology and enzymatic technique has endowed green catalytic synthesis with greater sustainable value, enhanced the reaction process, decreased reaction times and increased the productivity. This research provides a new technique for rapid synthesis of anti-cancer and antiviral nucleoside drugs and is helpful for further screening of drug activity. With this approach, more nucleoside esters can be efficiently synthesized.

## Author contributions

S.-Y. Z., G.-N. F. and L.-H. D.: subject selection, experimental design, drafted and revised the manuscript; S.-Y. Z., G.-N. F., Z.-K. S.: background research and experimental optimization; S.-Y. Z., Z.-K. S., A.-Y. Z., H.-J. X. and H. L.: collecting data; B.-L. Y., M.-M. X., Z.-X. R., G.-N. F., B.-L. P., T.-Y. Z. and X.-P. L.: analyze the data and revise the manuscript. All authors read and approved the final manuscript.

## Conflicts of interest

The authors declare that they have no known competing financial interests or personal relationships that could have appeared to influence the work reported in this paper.

## Supplementary Material

RA-014-D4RA00097H-s001
