# Supplementary material for: Continuous flow biocatalysis: synthesis of purine nucleoside esters catalyzed by lipase TL IM from Thermomyces lanuginosus
Source: RSC Adv. 2024 Apr 4;14(16):10953–61. doi: 10.1039/d4ra00097h (PMC10993233; doi:10.1039/d4ra00097h)
Supplement: RA-014-D4RA00097H-s001 [file RA-014-D4RA00097H-s001.pdf]

Electronic Supplementary Material (ESI) for RSC Advances.
This journal is © The Royal Society of Chemistry 2024

Supporting Information for

Continuous flow biocatalysis: synthesis of purine nucleoside esters catalyzed by lipase TL IM from *Thermomyces lanuginosus*

Shi-Yi Zhang,[a,#] Guo-Neng Fu,[a,#] Li-Hua Du,[a,*] Hang Lin,[a] Ao-Ying Zhang,[a] Han-Jia Xie,[a] Zhi-Kai Sheng,[a] Miao-miao Xue,[a] Bing-lin Yan,[a] Yi Liu,[a] Zhi-Xuan Ruan,[a] Bing-Le Pan,[a] Tong-Yao Zhou[a] and Xi-Ping Luo [b,*]

[a]College of Pharmaceutical Science, ZheJiang University of Technology, Zhejiang, Hangzhou, 310014, People's Republic of China

[b]Zhejiang Provincial Key Laboratory of Chemical Utilization of Forestry Biomass, Zhejiang A&F University, Zhejiang, Hangzhou, 311300, China

*Corresponding author. Fax: +86 18969069399. E-mail: orgdlh@zjut.edu.cn; orgdlh@gmail.com; luoxiping@zafu.edu.cn

[#] These authors contributed equally to this work and should be considered co-first authors.

Materials

All chemicals in this study were obtained from commercial sources and did not require further purification. Lipozyme TL IM form Thermomyces lanuginosus was purchased from Novo Nordisk. Adenosine was purchased from Macklin (Shanghai, China), Inosine was purchased from Accela (Shanghai, China), 6-chloropurine nucleoside was purchased from Aladdin (Shanghai, China). Vinyl acetate was purchased from SCRC (Shanghai, China), vinyl laurate from Aladdin (Shanghai, China), vinyl palmitate and divinyl adipate from TCI (Tokyo, Japan). Harvard Instrument PHD 2000 syringe pump was purchased from Harvard University (Holliston, MA, USA). The flow reactor and Y-mixer were purchased from Beijing Haigui Medical Engineering Design Co., Ltd. (Beijing China). A 400 MHz NMR spectrometer (Billerica, MA, USA) were also used in this study.

Purification of the product

When the conversion of the purine nucleoside esters reaches a maximum (determined by TLC), the reaction is terminated by filtering the enzyme, and the *tert*-amyl alcohol solvent is rotary evaporated

under reduced pressure. The product is separated by silica gel chromatography (eluent: dichloromethane/methanol from 30/1 to 24/1). Purification was monitored by TLC. The graded fractions containing the major product were combined, the solvent evaporated and the residue analyzed by ^{1}H NMR, ^{13}C NMR.

Experimental setup

A continuous-flow protocol for the enzymatic synthesis of purine nucleoside esters from purine nucleosides and vinyl esters in microreactors is described in Figure S1. T The experimental Apparatus consisted of five main components: a syringe pump (Harvard Apparatus Dr. 2000), substrate injectors, a Y-mixer, a flow reactor, and a product collector. Preparation: the flow reactor with an inner diameter of 2 mm was first filled with 0.870 g Lipozyme® TL IM, particle size 0.3-1.0 mm, reactivity 250 IUN·g $^{-1}$, and then immersed in a constant temperature water bath at 50 °C. Work began: 5.0 mmol purine riboside dissolved in 10 mL *tert*-amyl alcohol in feed 1 and 25.0 mmol vinyl esters dissolved in 10 mL *tert*-amyl alcohol in feed 2. The two solutions were intersected in a Y-type mixer, and the mixed flow was passed through the flow reactor at the flow rate of 18.3 uL min^{-1} with a residence time of 35 min. Finally, the reaction solution was collected and dried by evaporation. The products were separated by silica gel chromatography (eluent: dichloromethane/methanol from 30/1 to 24/1). The main products were determined by ^{1}H NMR and ^{13}C NMR.

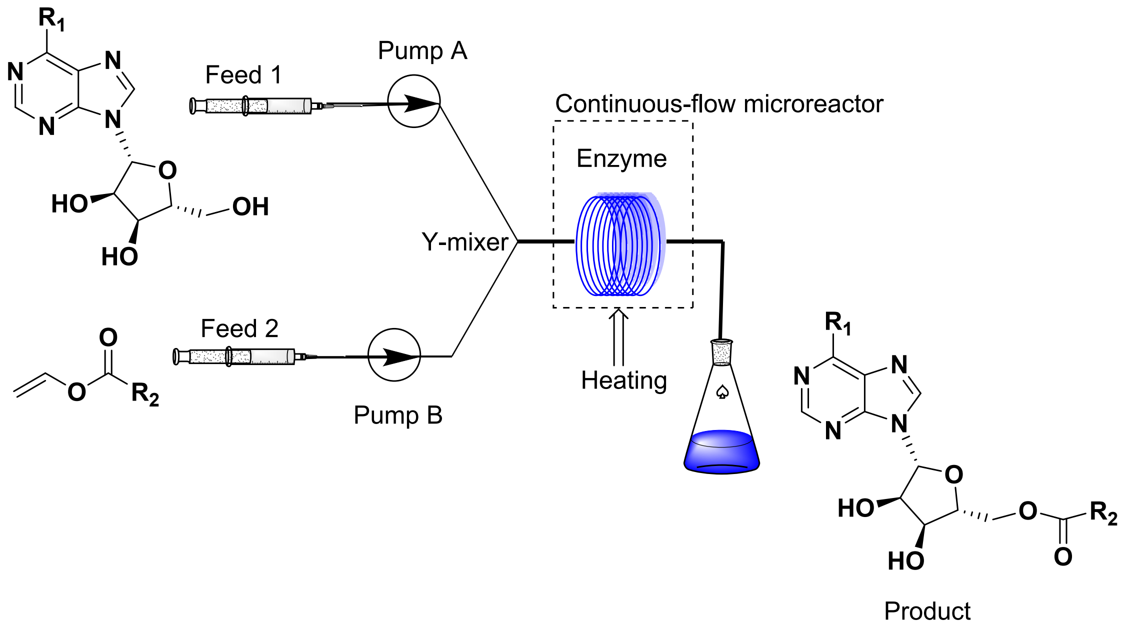

Figure S1. Equipment for the enzymatic synthesis of purine nucleoside esters from purine

nucleosides and vinyl esters catalyzed by Lipozyme TL IM from *Thermomyces lanuginosus* in continuous-flow microreactors.

Thin-Layer Chromatography

TLC analysis with methanol/dichloromethane 1/10 (v/v) as the eluent. The results were detected by UV irradiation at 254 nm.

Experiments

General procedure for the synthesis of purine nucleoside esters in a continuous flow microreactor

Silica gel tubes were filled with lipase TL IM and immersed in a constant temperature water bath to control the temperature. 5.0 mmol purine riboside dissolved in 10 mL tert-amyl alcohol in feed 1 and 25.0 mmol vinyl esters dissolved in 10 mL tert-amyl alcohol in feed 2. Feeds 1 and 2 were placed in separate 10 mL feeders and mixed together at a flow rate of 18.3 μL min^{-1} in a Y-mixer at 50 °C. The resulting stream (18.3 μL min^{-1}) was connected to a sample vial for collection of the final mixture. The final mixture was then evaporated and the residue separated by silica gel chromatography (200-300 mesh). Grades containing the major product were combined and the solvent was evaporated. The main products were determined by ^{1}H NMR and ^{13}C NMR.

Exploration of Reaction

Table 1. Batch and continuous-flow synthesis of purine nucleoside esters catalyzed by lipase TL IM from *Thermomyces lanuginosus*.

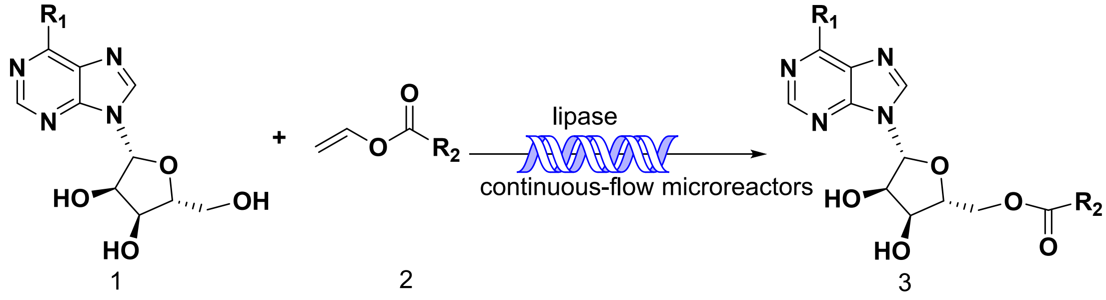

Entry	R_1	R_2	Method [a]	Time	Yield [b] (%)
1	NH_2	CH_3	**A**	26 h	59.1 ± 0.7(3a)
			**B**	35 min	64.0 ± 1.1(3a)

2	NH_2	$C_{11}H_{23}$	**A**	26 h	64.5 ± 0.6(3b)
			**B**	35 min	78.4 ± 0.9(3b)
3	NH_2	$C_{15}H_{31}$	**A**	26 h	63.1 ± 0.4(3c)
			**B**	35 min	79.2 ± 1.6(3c)
4	NH_2	$(CH_2)_4COOCH{=}CH_2$	**A**	26 h	60.6 ± 0.5(3d)
			**B**	35 min	74.7 ± 0.7(3d)
5	OH	CH_3	**A**	26 h	58.3 ± 0.9(3e)
			**B**	35 min	67.5 ± 0.6(3e)
6	OH	$C_{11}H_{23}$	**A**	26 h	77.4 ± 0.7(3f)
			**B**	35 min	86.1 ± 1.2(3f)
7	OH	$C_{15}H_{31}$	**A**	26 h	78.6 ± 1.3(3g)
			**B**	35 min	88.7 ± 0.9(3g)
8	OH	$(CH_2)_4COOCH{=}CH_2$	**A**	26 h	73.2 ± 0.7(3h)
			**B**	35 min	77.3 ± 1.4(3h)
9	Cl	CH_3	**A**	26 h	61.7 ± 0.7(3i)
			**B**	35 min	71.4 ± 0.8(3i)
10	Cl	$C_{11}H_{23}$	**A**	26 h	85.3 ± 1.3(3j)
			**B**	35 min	93.7 ± 0.9(3j)
11	Cl	$C_{15}H_{31}$	**A**	26 h	84.6 ± 0.8(3k)
			**B**	35 min	92.9 ± 1.1(3k)
12	Cl	$(CH_2)_4COOCH{=}CH_2$	**A**	26 h	72.3 ± 0.8(3l)
			**B**	35 min	80.5 ± 1.6(3l)

[a] Method A: batch reactor, 5.0 mmol purine nucleoside (1) and 25.0 mmol vinyl esters (2) added to 20 mL *tert*-amyl alcohol in a 50 mL erlenmeyer flask, 0.870 g lipase TL IM M (catalyst reactivity: 250IUN·g^{-1}), 26 h, 50 °C. Method B: continuous-flow reactor, feed 1, 10 mL *tert*-amyl alcohol contained 5.0 mmol purine nucleoside (1); feed 2, 10 mL *tert*-amyl alcohol contained 25.0 mmol vinyl esters (2), lipase TL IM 0.870 g (catalyst reactivity: 250IUN·g^{-1}), flow rate 18.3 μL min^{-1}, residence time 35 min, 50 °C. [b] Isolated yield. Yield: 100 × (actual received quantity/ideal calculated quantity). The data are presented as average ± SD of triplicate experiments.

Experimental data of products

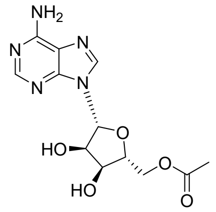

(5-(6-amino-9H-purin-9-yl)-3,4-dihydroxytetrahydrofuran-2-yl)methyl acetate (**3a**). White solid, ^{1}H NMR (400 MHz, DMSO-d_6) δ 8.32 (s, 1H), 8.16 (d, J = 3.6 Hz, 1H), 7.33 (s, 2H), 5.93 (s, 1H), 5.61 (s, 1H), 5.43 – 5.35 (m, 1H), 4.69 (s, 1H), 4.18 (d, J = 80.4 Hz, 4H), 2.01 (s, 3H).; ^{13}C NMR (101 MHz, DMSO) δ 170.68, 156.54, 153.14, 149.80, 140.20, 119.60, 88.19, 81.94, 73.29, 70.75, 64.37, 21.05.

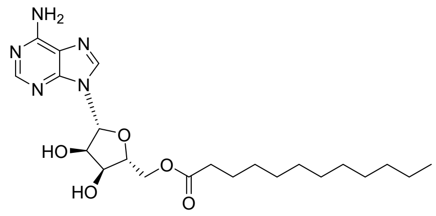

(5-(6-amino-9H-purin-9-yl)-3,4-dihydroxytetrahydrofuran-2-yl)methyl dodecanoate (**3b**). White solid, ^{1}H NMR (400 MHz, DMSO-d_6) δ 8.30 (s, 1H), 8.14 (s, 1H), 7.30 (s, 2H), 5.90 (d, J = 4.9 Hz, 1H), 5.59 (d, J = 5.6 Hz, 1H), 5.38 (d, J = 5.4 Hz, 1H), 4.66 (q, J = 5.2 Hz, 1H), 4.33 (dd, J = 11.9, 3.7 Hz, 1H), 4.29 – 4.23 (m, 1H), 4.19 (dd, J = 11.9, 6.1 Hz, 1H), 4.11 – 4.03 (m, 1H), 2.32 – 2.24 (m, 2H), 1.51 – 1.43 (m, 2H), 1.21 (d, J = 5.8 Hz, 16H), 0.85 (t, J = 6.7 Hz, 3H).; ^{13}C NMR (101 MHz, DMSO) δ 173.26, 156.54, 153.11, 149.93, 140.14, 119.59, 88.22, 81.92, 73.34, 70.70, 64.14, 33.77, 31.75, 29.43, 29.31, 29.16, 29.11, 28.84, 24.83, 22.56, 14.43.

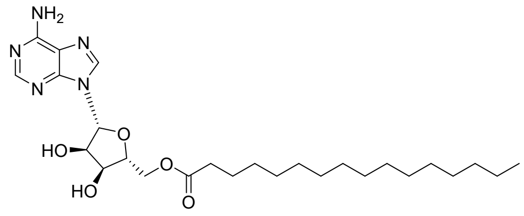

(5-(6-amino-9H-purin-9-yl)-3,4-dihydroxytetrahydrofuran-2-yl)methyl palmitate (**3c**). White solid, ^{1}H NMR (400 MHz, DMSO-d_6) δ 8.31 (s, 1H), 8.14 (s, 1H), 7.33 (s, 2H), 5.91 (d, J = 4.8 Hz, 1H), 5.59 (d, J = 5.8 Hz, 1H), 5.38 (d, J = 5.5 Hz, 1H), 4.66 (q, J = 5.2 Hz, 1H), 4.37 – 4.14 (m, 3H), 4.07 (q, J = 5.2, 4.8 Hz, 1H), 2.28 (t, J = 7.4 Hz, 2H), 1.47 (p, J = 6.7 Hz, 2H), 1.29 – 1.15 (m, 24H), 0.85 (t, J = 6.6 Hz, 3H).; ^{13}C NMR (101 MHz, DMSO) δ 173.22, 156.54, 153.07, 149.77, 140.13, 119.62, 88.23, 81.91, 73.35, 70.73, 64.18, 33.77, 31.77, 29.52, 29.49, 29.44, 29.34, 29.19, 29.15, 28.87, 24.84, 22.57, 14.42.

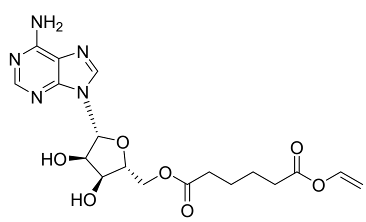

(5-(6-amino-9H-purin-9-yl)-3,4-dihydroxytetrahydrofuran-2-yl)methyl vinyl adipate (**3d**). Yellowish solid, ^{1}H NMR (400 MHz, DMSO-d_6) δ 8.32 (s, 1H), 8.16 (s, 1H), 7.32 (s, 2H), 7.21 (t, J = 10.2 Hz, 1H), 5.92 (m, 1H), 4.89 (s, 1H), 4.67 (s, 2H), 4.38 – 4.31 (m, 1H), 4.29 (s, 1H), 4.22 (s, 1H), 4.07 (s, 1H), 2.86 (s, 1H), 2.42 (s, 1H), 2.28 (s, 3H), 2.17 (s, 1H), 1.16 (d, J = 49.4 Hz, 4H).; ^{13}C NMR (101 MHz, DMSO) δ 173.68, 170.75, 156.53, 153.10, 149.78, 141.65, 140.17, 119.60, 98.35, 88.28, 81.93, 73.39, 70.74, 64.24, 34.09, 33.46, 24.46, 24.22.

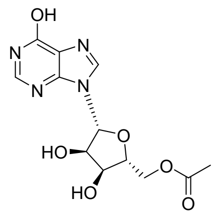

(3,4-dihydroxy-5-(6-hydroxy-9H-purin-9-yl)tetrahydrofuran-2-yl)methyl acetate (**3e**). White solid, ^{1}H NMR (400 MHz, DMSO-d_6) δ 12.43 (s, 1H), 8.29 (s, 1H), 8.09 (s, 1H), 5.91 (d, *J* = 5.0 Hz, 1H), 5.65 (s, 1H), 4.58 (t, *J* = 5.1 Hz, 1H), 4.31 (dd, *J* = 11.9, 3.6 Hz, 1H), 4.19 (dq, *J* = 11.8, 6.0, 5.5 Hz, 2H), 4.10 (dt, *J* = 5.8, 3.9 Hz, 1H), 3.17 (s, 1H), 2.02 (s, 3H).; ^{13}C NMR (101 MHz, DMSO-d_6) δ 170.70, 157.06, 148.70, 146.43, 139.34, 124.95, 88.15, 82.17, 73.75, 70.70, 64.30, 21.05.

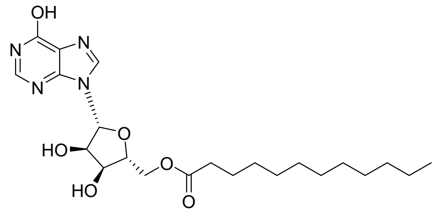

(3,4-dihydroxy-5-(6-hydroxy-9H-purin-9-yl)tetrahydrofuran-2-yl)methyl dodecanoate (**3f**). White solid, ^{1}H NMR (400 MHz, DMSO-d_6) δ 12.42 (s, 1H), 8.27 (s, 1H), 8.07 (s, 1H), 5.89 (d, *J* = 4.9 Hz, 1H), 5.63 (d, *J* = 5.8 Hz, 1H), 5.43 – 5.38 (m, 1H), 4.55 (q, *J* = 4.3 Hz, 1H), 4.31 (dd, *J* = 12.0, 3.7 Hz, 1H), 4.19 (q, *J* = 6.1 Hz, 2H), 4.09 (q, *J* = 4.8 Hz, 1H), 2.29 (t, *J* = 7.4 Hz, 2H), 1.48 (p, *J* = 6.9 Hz, 2H), 1.30 – 1.18 (m, 16H), 0.85 (s, 2H), 0.84 (d, *J* = 13.4 Hz, 1H). ^{13}C NMR (101 MHz, DMSO) δ 173.23, 157.02, 148.66, 146.37, 139.23, 124.97, 88.17, 82.12, 73.83, 70.67, 64.12, 33.78, 31.76, 29.45, 29.34, 29.18, 29.15, 28.86, 24.86, 22.57, 14.42.

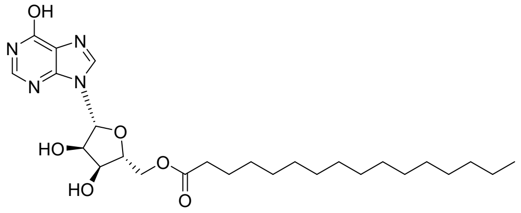

(3,4-dihydroxy-5-(6-hydroxy-9H-purin-9-yl)tetrahydrofuran-2-yl)methyl palmitate (**3g**). White solid. ^{1}H NMR (400 MHz, DMSO-d_6) δ 8.26 (s, 1H), 8.06 (s, 1H), 5.88 (d, *J* = 4.9 Hz, 1H), 5.65 – 5.60 (m, 1H), 5.40 (s, 1H), 4.54 (t, *J* = 5.0 Hz, 1H), 4.30 (dd, *J* = 11.9, 3.7 Hz, 1H), 4.23 – 4.14 (m, 2H), 4.12 – 4.04 (m, 1H), 2.50 (t, *J* = 2.0 Hz, 1H), 2.29 (t, *J* = 7.3 Hz, 2H), 1.47 (q, *J* = 7.1 Hz, 2H), 1.35 – 1.18 (m, 24H), 0.84 (t, *J* = 6.7 Hz, 3H). ^{13}C NMR (101 MHz, Chloroform-*d*) δ 172.78, 156.58, 148.22, 145.93, 138.79, 124.53, 87.74, 81.68, 73.39, 70.23, 63.69, 33.34, 31.33, 29.08, 29.05, 29.01, 28.91, 28.75, 28.72, 28.60, 28.44, 24.42, 22.13, 13.97.

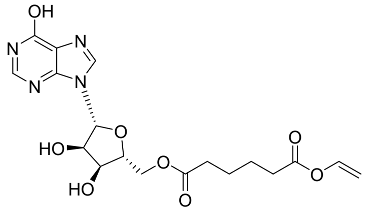

(3,4-dihydroxy-5-(6-hydroxy-9H-purin-9-yl)tetrahydrofuran-2-yl)methyl vinyl adipate (**3h**). White solid, ^{1}H NMR (400 MHz, DMSO-d_6) δ 12.42 (s, 1H), 8.28 (s, 1H), 8.09 (s, 1H), 7.20 (dd, *J* = 14.0, 6.3 Hz, 1H), 5.91 (d, *J* = 4.8 Hz, 1H), 5.84 – 5.14 (m, 1H), 4.89 (d, *J* = 1.6 Hz, 1H), 4.85 (d, *J* = 1.6 Hz, 1H), 4.66 – 4.54 (m, 2H), 4.32 (dd, *J* = 11.9, 3.7 Hz, 1H), 4.25 – 4.16 (m, 2H), 4.11 (dt, *J* = 5.8, 4.0 Hz, 1H), 2.46 – 2.38 (m, 2H), 2.38 – 2.30 (m, 2H), 1.55 (tt, *J* = 7.2, 3.7 Hz, 4H).; ^{13}C NMR (101 MHz, DMSO-d_6) δ 173.03, 170.69, 157.07, 148.68, 146.41, 141.65, 139.30, 124.96, 98.47, 88.19, 82.14, 73.79, 70.68, 64.20, 33.35, 33.09, 24.11, 23.86.

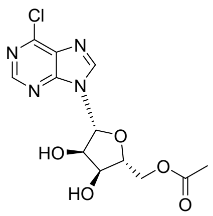

(5-(6-chloro-9H-purin-9-yl)-3,4-dihydroxytetrahydrofuran-2-yl)methyl acetate (**3i**). White solid, ^{1}H NMR (400 MHz, DMSO-d_6) δ 8.87 (s, 1H), 8.81 (s, 1H), 6.07 (d, J = 4.6 Hz, 1H), 5.71 (d, J = 5.7 Hz, 1H), 5.48 (d, J = 5.5 Hz, 1H), 4.71 (q, J = 5.1 Hz, 1H), 4.38 – 4.12 (m, 4H), 2.01 (s, 3H).; ^{13}C NMR (101 MHz, DMSO) δ 170.68, 152.24, 151.95, 149.88, 146.49, 131.92, 88.99, 82.35, 73.54, 70.62, 64.17, 21.02.

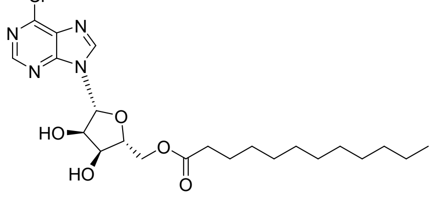

(5-(6-chloro-9H-purin-9-yl)-3,4-dihydroxytetrahydrofuran-2-yl)methyl dodecanoate (**3j**). White solid, ^{1}H NMR (400 MHz, DMSO-d_6) δ 8.86 (s, 1H), 8.80 (s, 1H), 6.07 (d, J = 4.5 Hz, 1H), 5.69 (d, J = 5.4 Hz, 1H), 5.45 (d, J = 5.6 Hz, 1H), 4.71 (q, J = 5.0 Hz, 1H), 4.32 (qd, J = 11.5, 11.1, 4.7 Hz, 2H), 4.26 – 4.20 (m, 1H), 4.16 (q, J = 4.9 Hz, 1H), 2.25 (td, J = 7.4, 2.4 Hz, 2H), 1.44 (p, J = 6.8 Hz, 2H), 1.30 – 1.15 (m, 16H), 0.81 (t, J = 6.7 Hz, 3H) .; ^{13}C NMR (101 MHz, DMSO) δ 173.12, 152.16, 151.93, 149.90, 146.46, 131.94, 89.07, 82.30, 73.62, 70.58, 63.90, 33.76, 31.75, 29.44, 29.33, 29.17, 29.13, 28.86, 24.82, 22.55, 14.31.

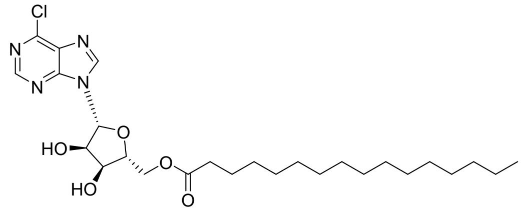

(5-(6-chloro-9H-purin-9-yl)-3,4-dihydroxytetrahydrofuran-2-yl)methyl palmitate (**3k**). White solid, ^{1}H NMR (400 MHz, DMSO-d_6) δ 8.87 (s, 1H), 8.82 (s, 1H), 6.06 (d, J = 4.5 Hz, 1H), 5.68 (d, J = 5.4 Hz, 1H), 5.44 (d, J = 5.6 Hz, 1H), 4.71 (q, J = 5.0 Hz, 1H), 4.38 – 4.19 (m, 3H), 4.14 (td, J = 5.6, 3.6 Hz, 1H), 3.33 (d, J = 1.8 Hz, 4H), 2.25 (td, J = 7.4, 2.8 Hz, 2H), 1.44 (p, J = 7.1 Hz, 2H), 1.22 (d, J = 3.5 Hz, 13H), 1.17 (s, 7H), 0.89 – 0.81 (m, 3H).; ^{13}C NMR (101 MHz, DMSO) δ 173.19, 152.24, 151.98, 149.88, 146.55, 131.94, 89.03, 82.32, 73.55, 70.56, 63.89, 33.76, 31.76, 29.51, 29.49, 29.45, 29.41, 29.30, 29.17, 29.09, 28.82, 24.83, 22.56, 14.40.

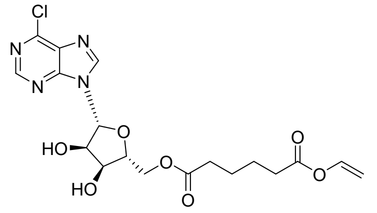

(5-(6-chloro-9H-purin-9-yl)-3,4-dihydroxytetrahydrofuran-2-yl)methyl vinyl adipate (**3l**). Yellow solid, ^{1}H NMR (400 MHz, DMSO-d_6) δ 8.81 (d, J = 22.4 Hz, 2H), 7.16 (dd, J = 14.0, 6.3 Hz, 1H), 6.08 (d, J = 4.4 Hz, 1H), 5.71 (d, J = 5.5 Hz, 1H), 5.47 (d, J = 5.6 Hz, 1H), 4.82 (dd, J = 14.0, 1.6 Hz, 1H), 4.73 (q, J = 5.0 Hz, 1H), 4.58 (dd, J = 6.4, 1.6 Hz, 1H), 4.32 (dtd, J = 29.1, 12.0, 4.7 Hz, 3H), 4.18 (td, J = 5.4, 3.5 Hz, 1H), 2.39 (t, J = 6.4 Hz, 2H), 2.31 (t, J = 5.3 Hz, 2H), 1.58 – 1.48 (m, 4H).; ^{13}C NMR (101 MHz, DMSO) δ 172.96, 170.57, 152.13, 151.89, 149.90, 146.36, 141.55, 131.92, 98.24, 89.07, 82.29, 73.64, 70.60, 64.01, 33.35, 33.08, 24.08, 23.82.

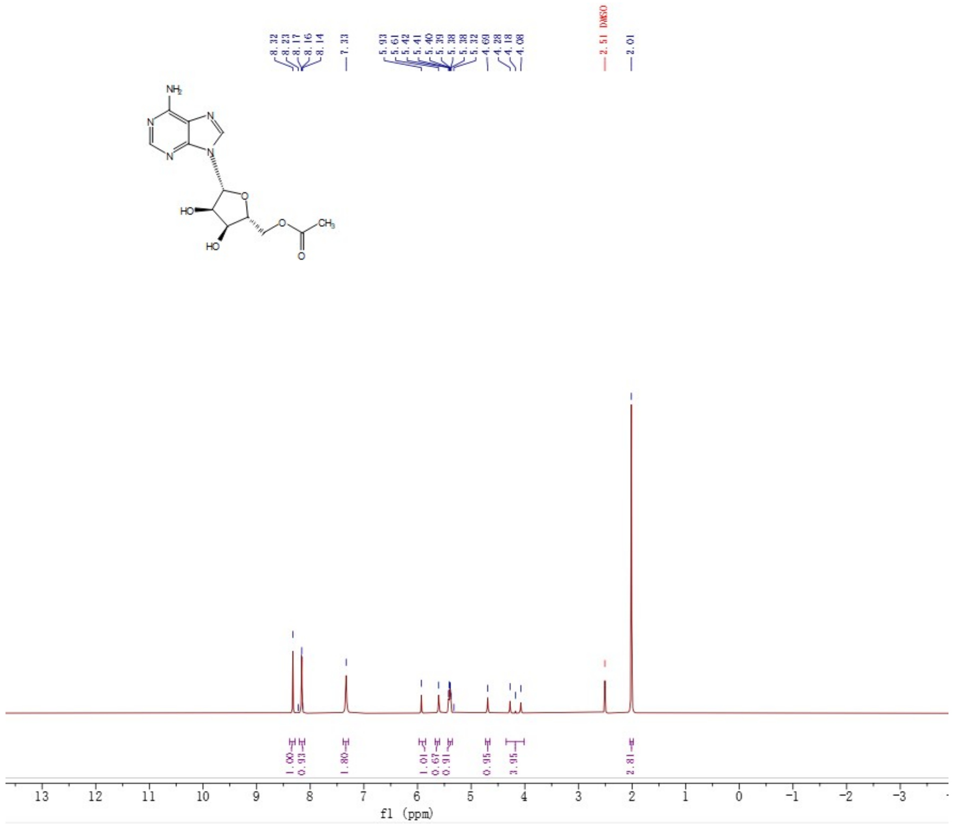
8.32
8.23
8.17
8.16
8.14
7.33
5.93
5.61
5.42
5.41
5.40
5.39
5.38
5.38
5.32
4.69
4.28
4.18
4.08
2.51 DMSO
2.01
NH2
N
O
HO
HO
CH3
1.00
0.93
1.80
1.01
0.67
0.91
0.95
3.95
2.81
f1 (ppm)

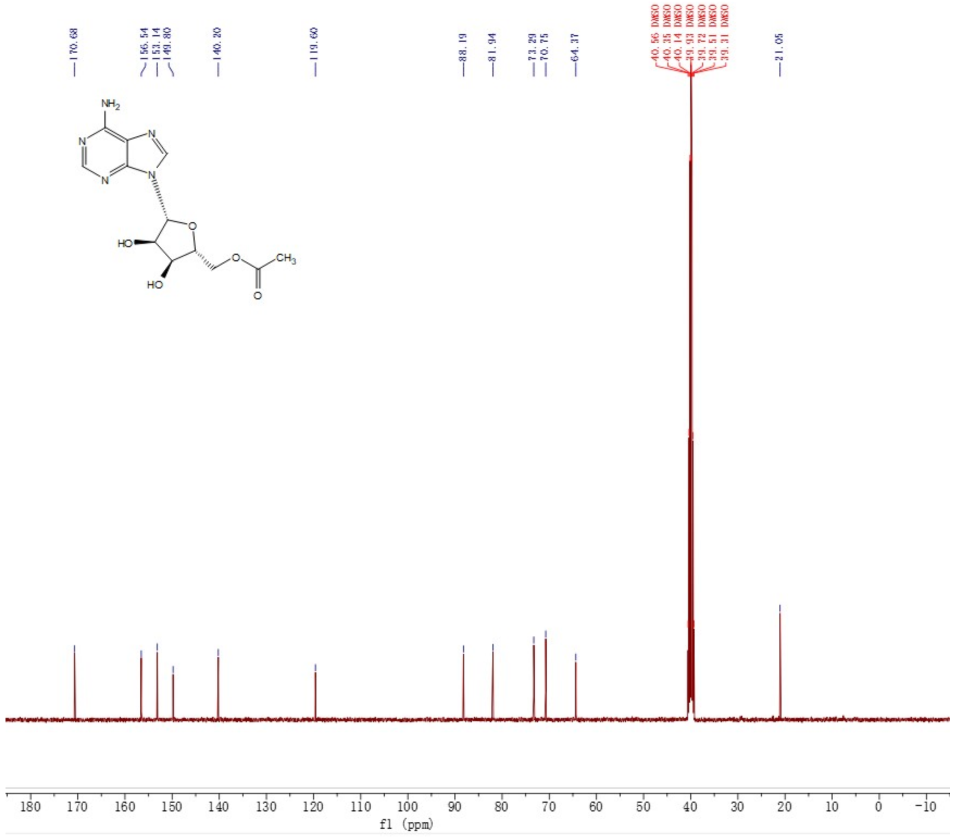
170.68
156.54
153.14
149.80
140.20
119.60
88.19
81.94
73.29
70.75
64.37
40.56 DMSO
40.35 DMSO
40.14 DMSO
39.93 DMSO
39.72 DMSO
39.51 DMSO
39.31 DMSO
21.05
NH2
N
O
HO
HO
CH3
f1 (ppm)

3b

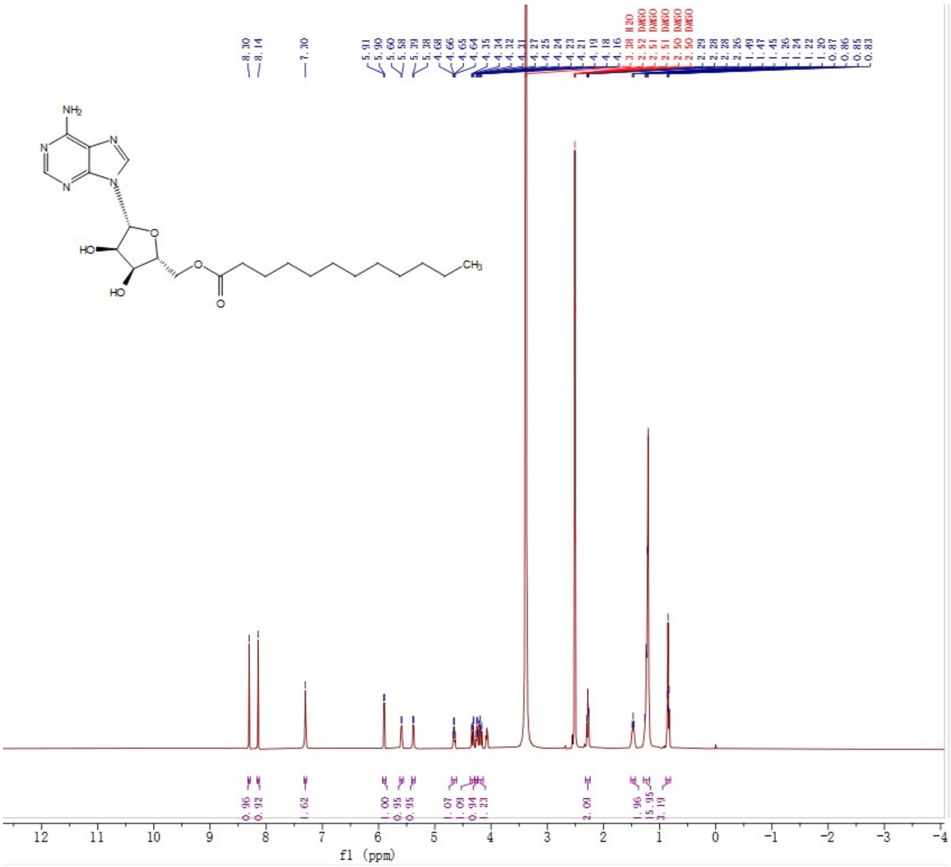

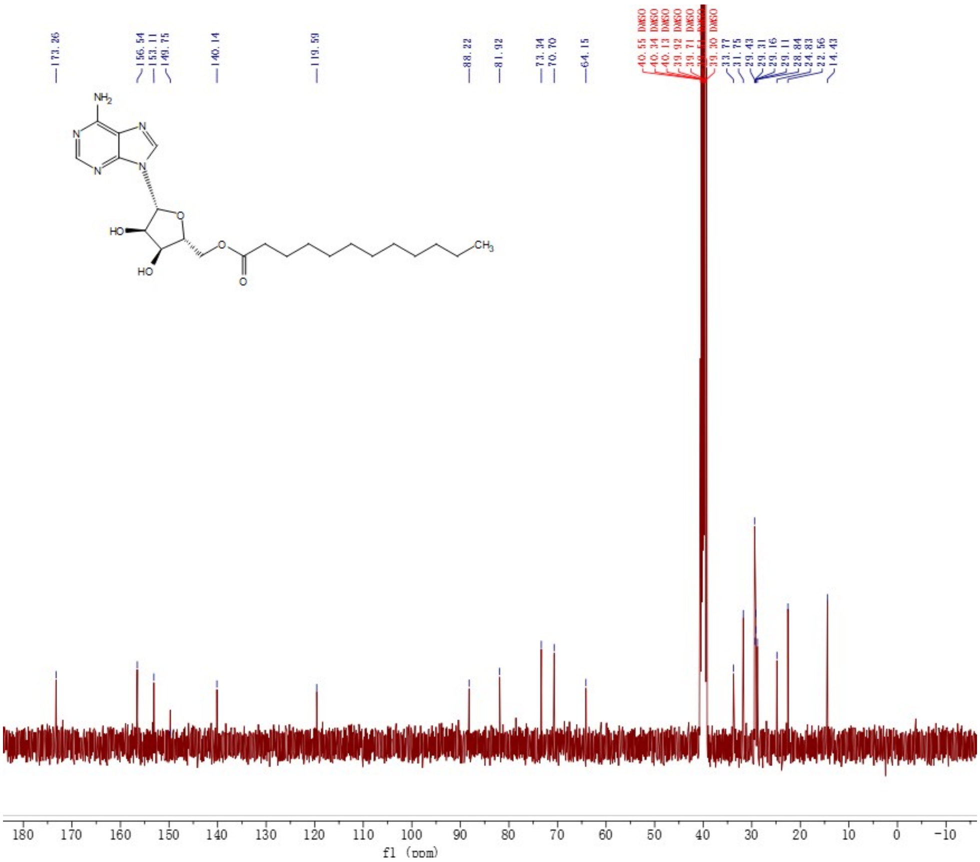

3c

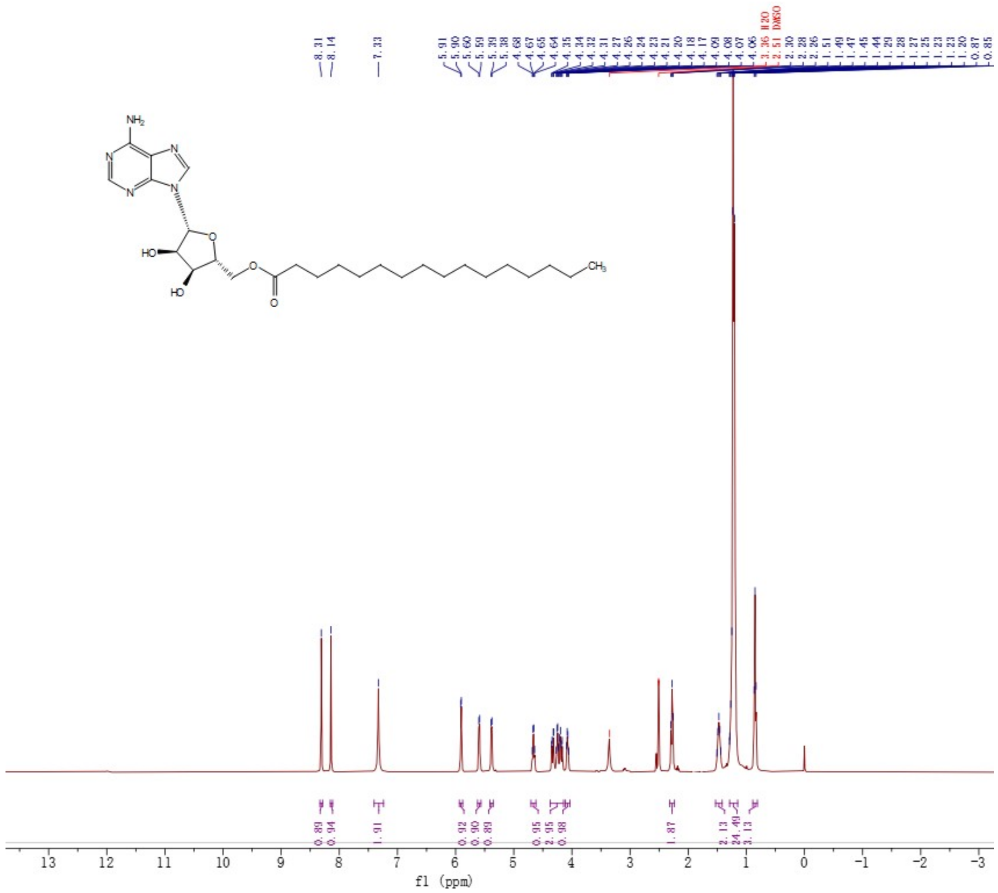
8.31
8.14
7.33
5.91
5.90
5.60
5.59
5.39
5.38
4.68
4.67
4.65
4.64
4.35
4.34
4.32
4.31
4.27
4.26
4.24
4.23
4.21
4.20
4.18
4.17
4.09
4.08
4.07
4.06
3.36 H2O
2.51 DMSO
2.30
2.28
2.26
1.51
1.49
1.47
1.45
1.44
1.29
1.28
1.27
1.25
1.23
1.23
1.20
0.87
0.85
NH2
N
HO
O
CH3
0.89
0.94
1.91
0.92
0.90
0.89
0.95
2.95
0.98
1.87
2.13
24.49
3.13
f1 (ppm)

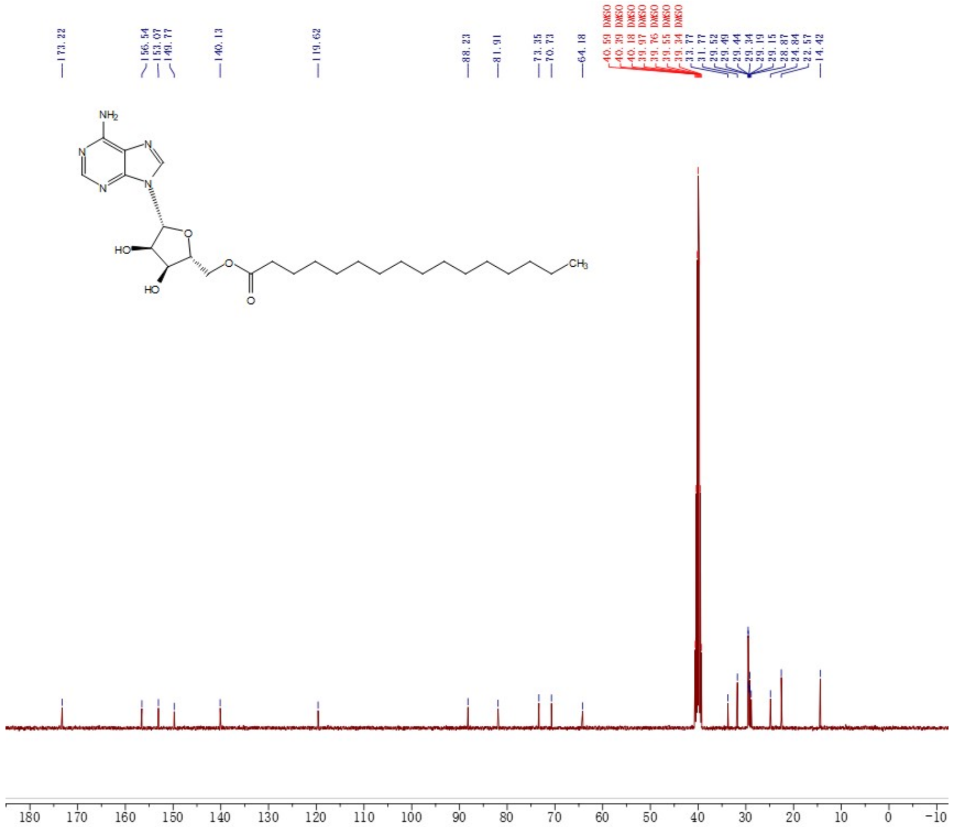
173.22
156.54
153.07
149.77
140.13
119.62
88.23
81.91
73.35
70.73
64.18
40.59 DMSO
40.39 DMSO
40.18 DMSO
39.97 DMSO
39.76 DMSO
39.55 DMSO
39.34 DMSO
33.77
31.77
29.52
29.49
29.44
29.34
29.19
29.15
28.87
24.84
22.57
14.42
NH2
N
HO
O
CH3
f1 (ppm)

3d

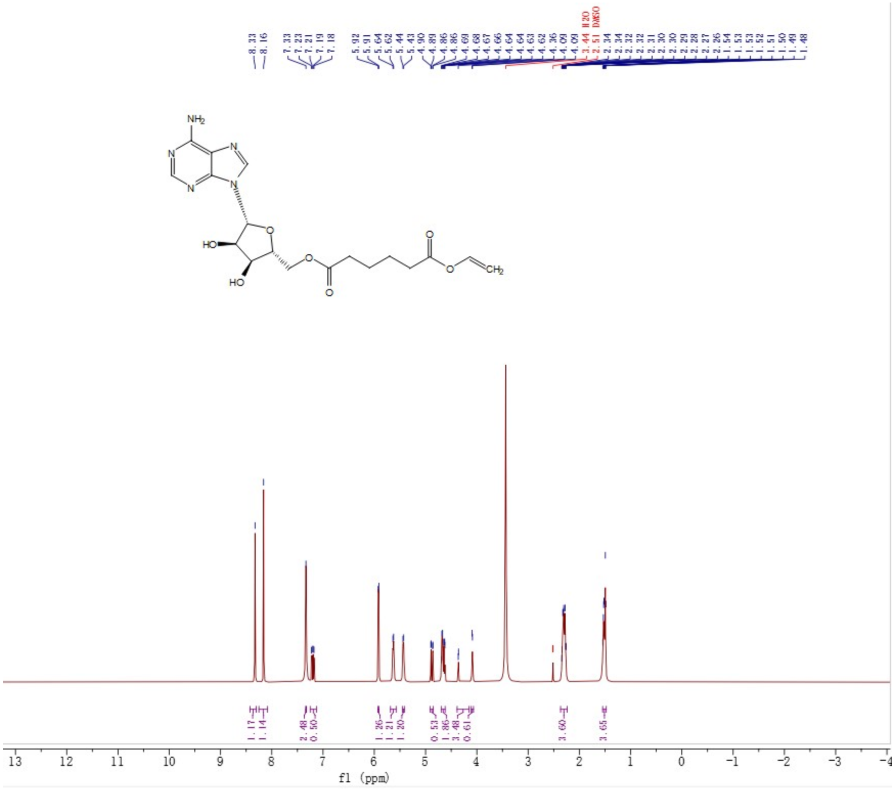
NH2
N
N
N
N
O
HO
HO
O
O
O
O
CH2
f1 (ppm)

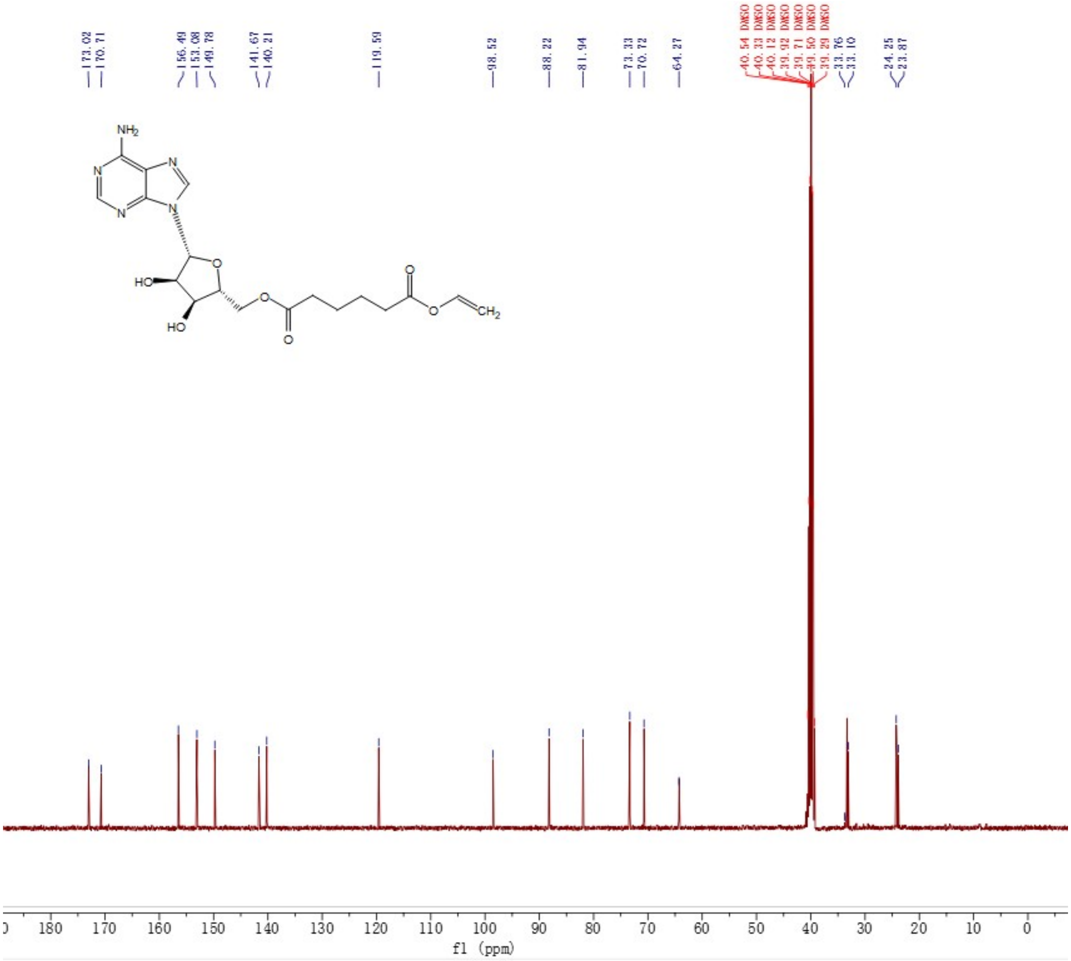
173.02
170.71
156.49
153.08
149.78
141.67
140.21
119.59
98.52
88.22
81.94
73.33
70.72
64.27
40.54 DMSO
40.33 DMSO
40.12 DMSO
39.92 DMSO
39.71 DMSO
39.50 DMSO
39.29 DMSO
33.76
33.10
24.25
23.87
NH2
N
N
N
N
O
HO
HO
O
O
O
O
CH2
f1 (ppm)

3e

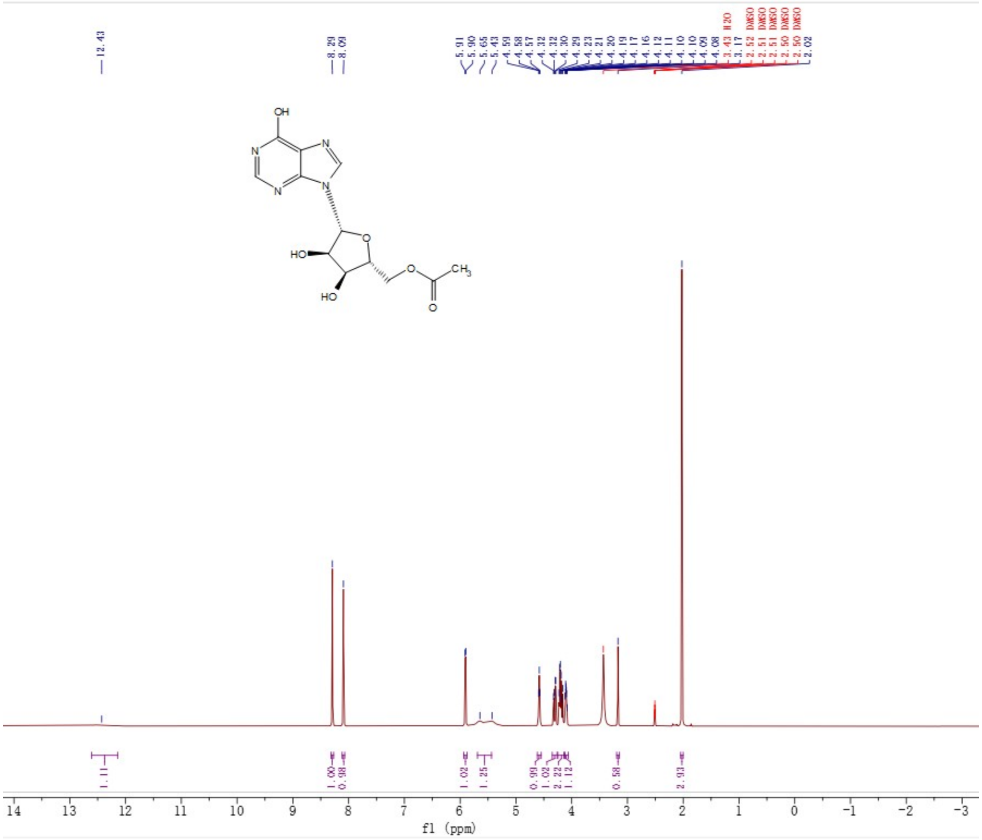
12.43
8.29
8.09
5.91
5.90
5.65
5.43
4.59
4.58
4.57
4.32
4.32
4.30
4.29
4.23
4.21
4.20
4.19
4.17
4.16
4.12
4.11
4.10
4.09
4.08
3.43 H2O
3.17
2.52 DMSO
2.51 DMSO
2.51 DMSO
2.50 DMSO
2.50 DMSO
2.02
OH
N
N
N
N
HO
HO
O
O
CH3
O
1.11
1.00
0.98
1.02
1.25
0.99
1.02
2.22
1.12
0.58
2.93
f1 (ppm)

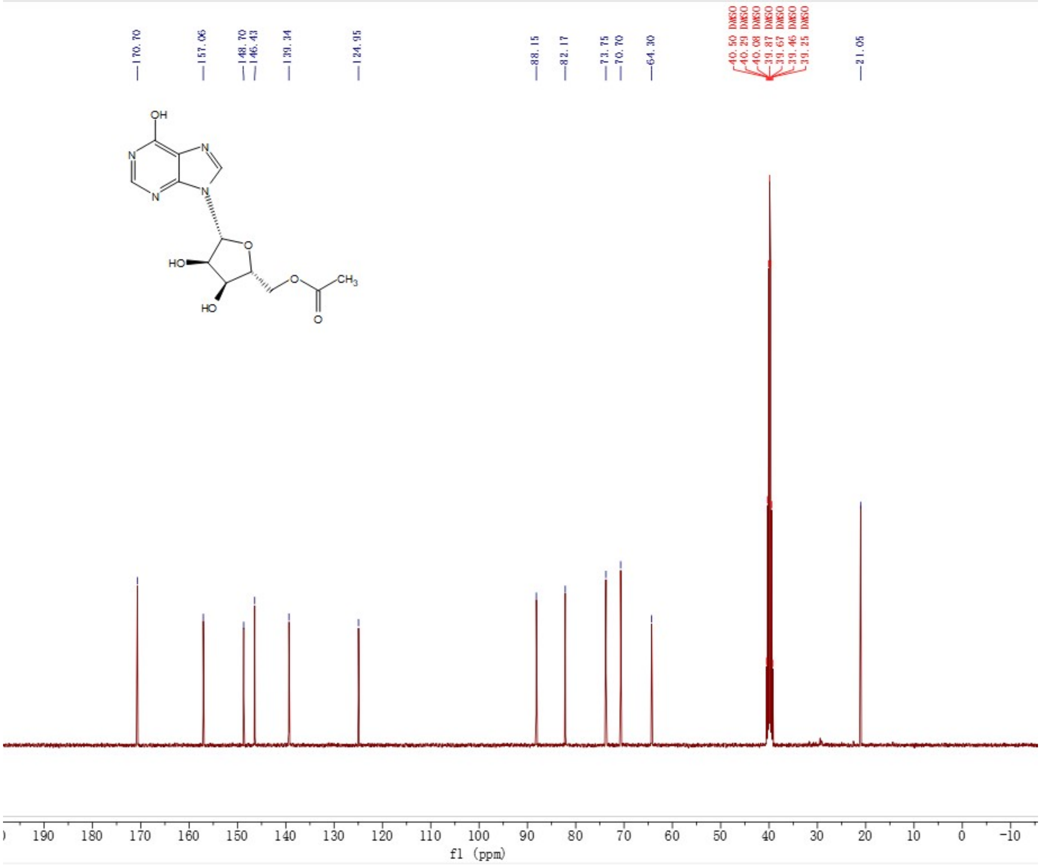
170.70
157.06
148.70
146.43
139.34
124.95
88.15
82.17
73.75
70.70
64.30
40.50 DMSO
40.29 DMSO
40.08 DMSO
39.87 DMSO
39.67 DMSO
39.46 DMSO
39.25 DMSO
21.05
OH
N
N
N
N
HO
HO
O
O
CH3
O
f1 (ppm)

3f

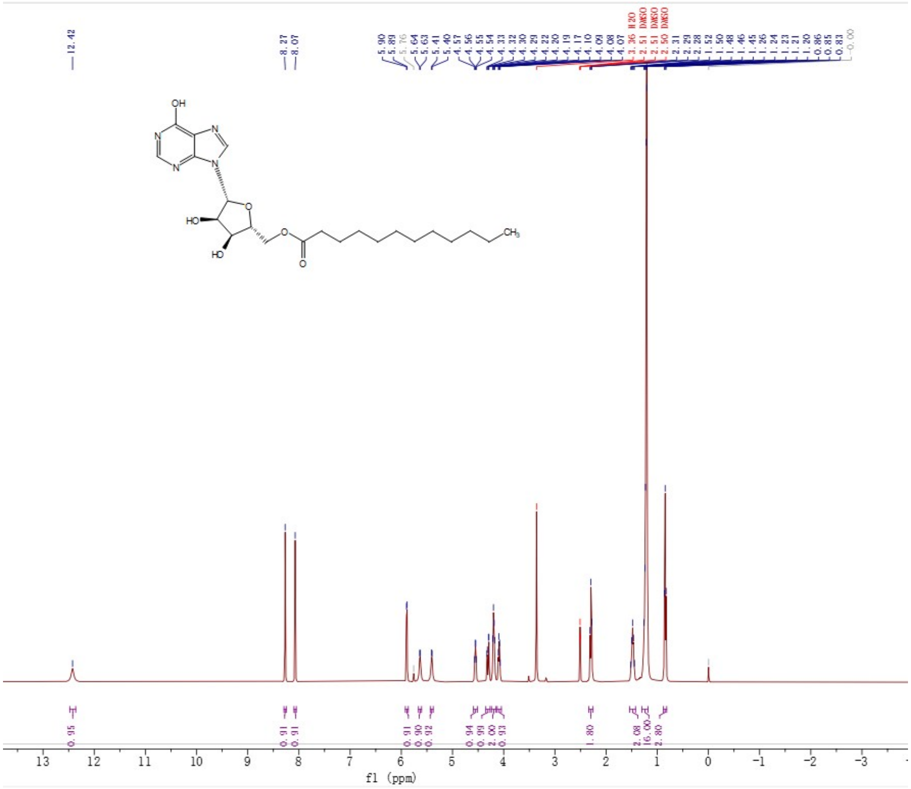

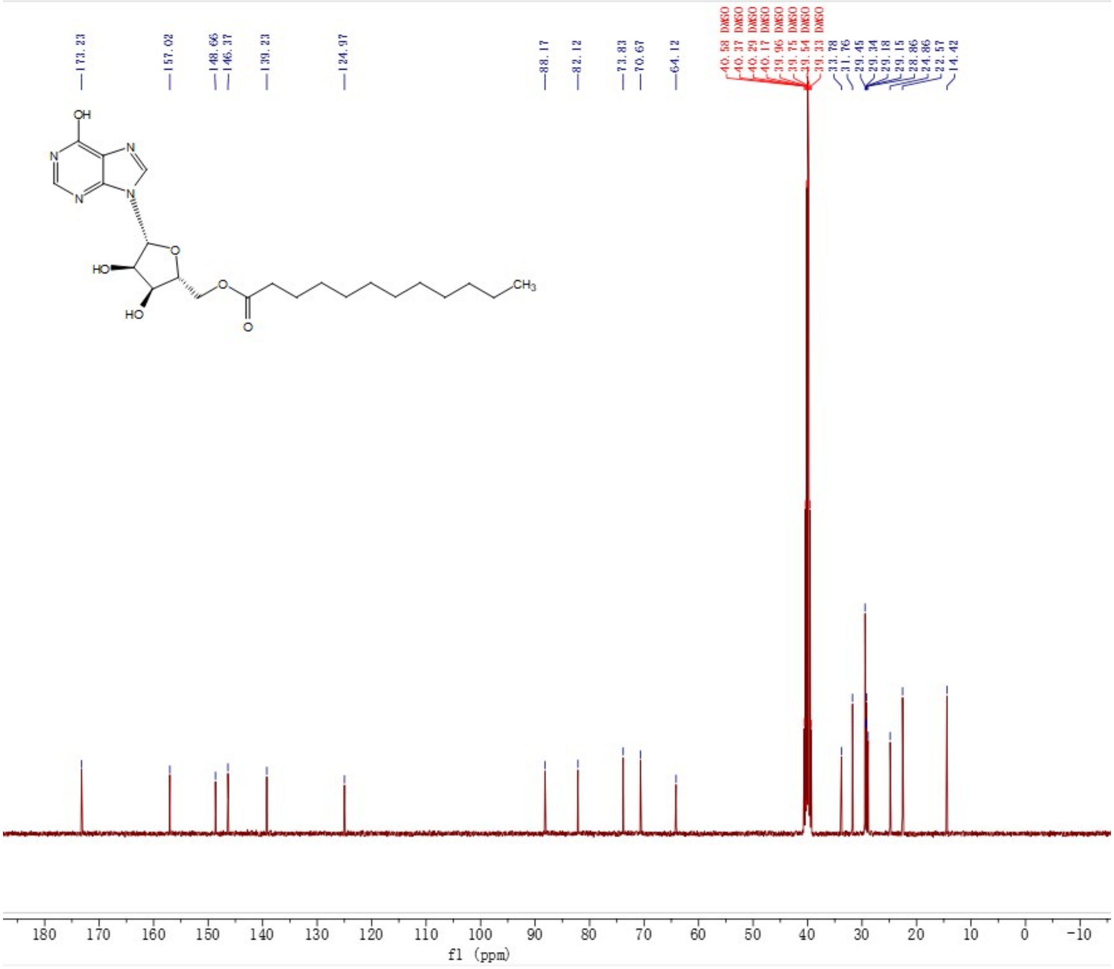

3g

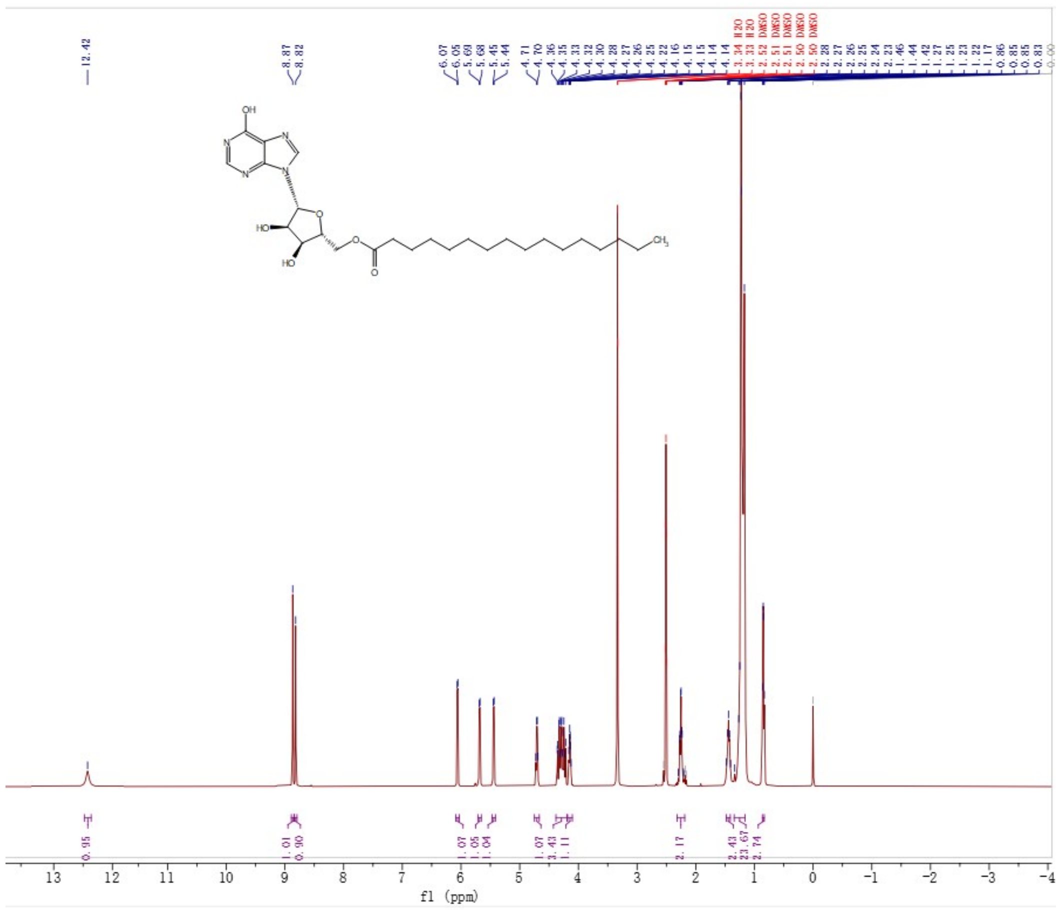

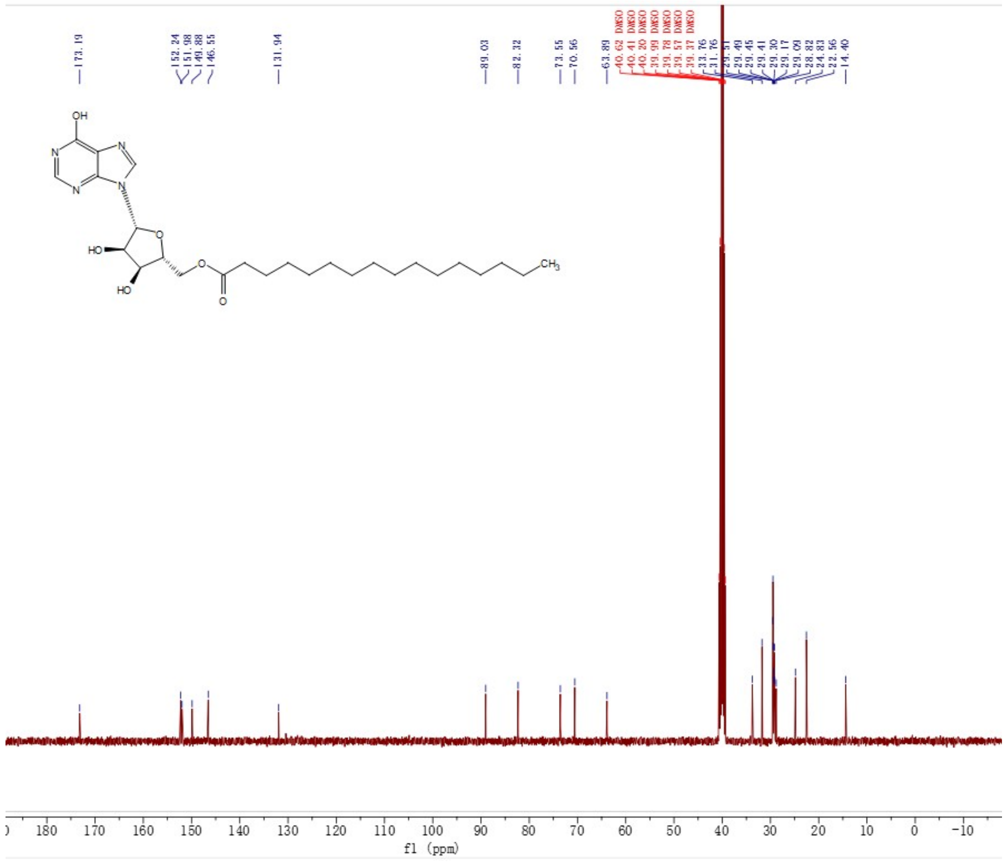

3h

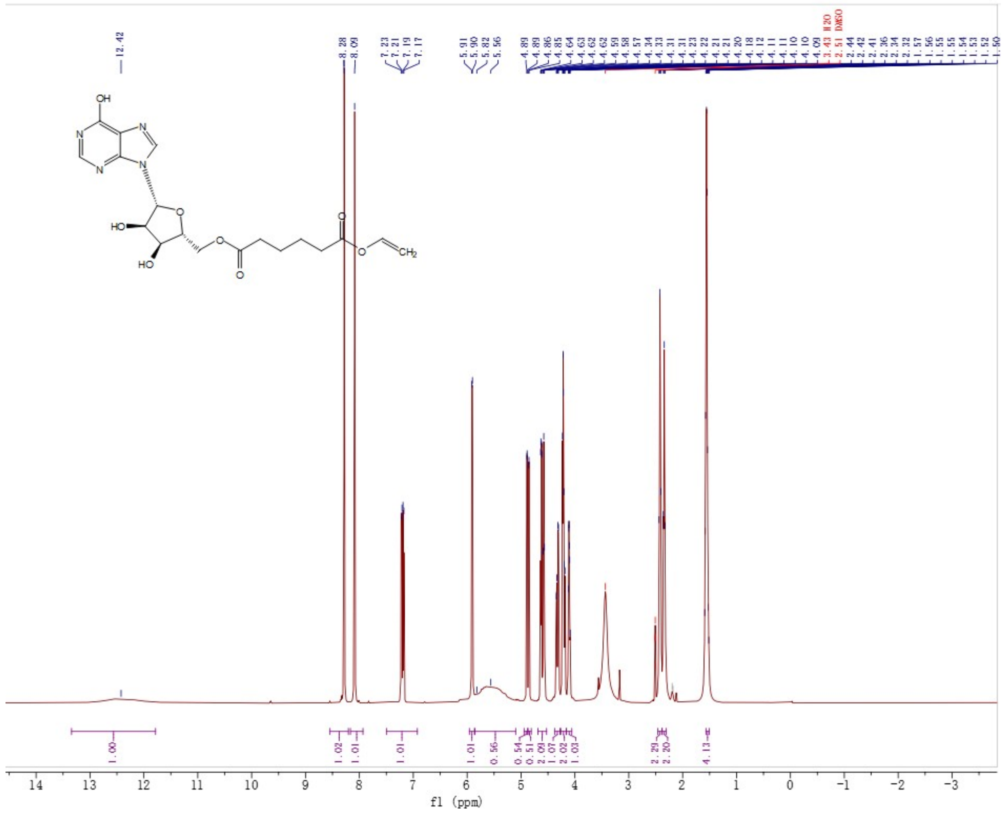

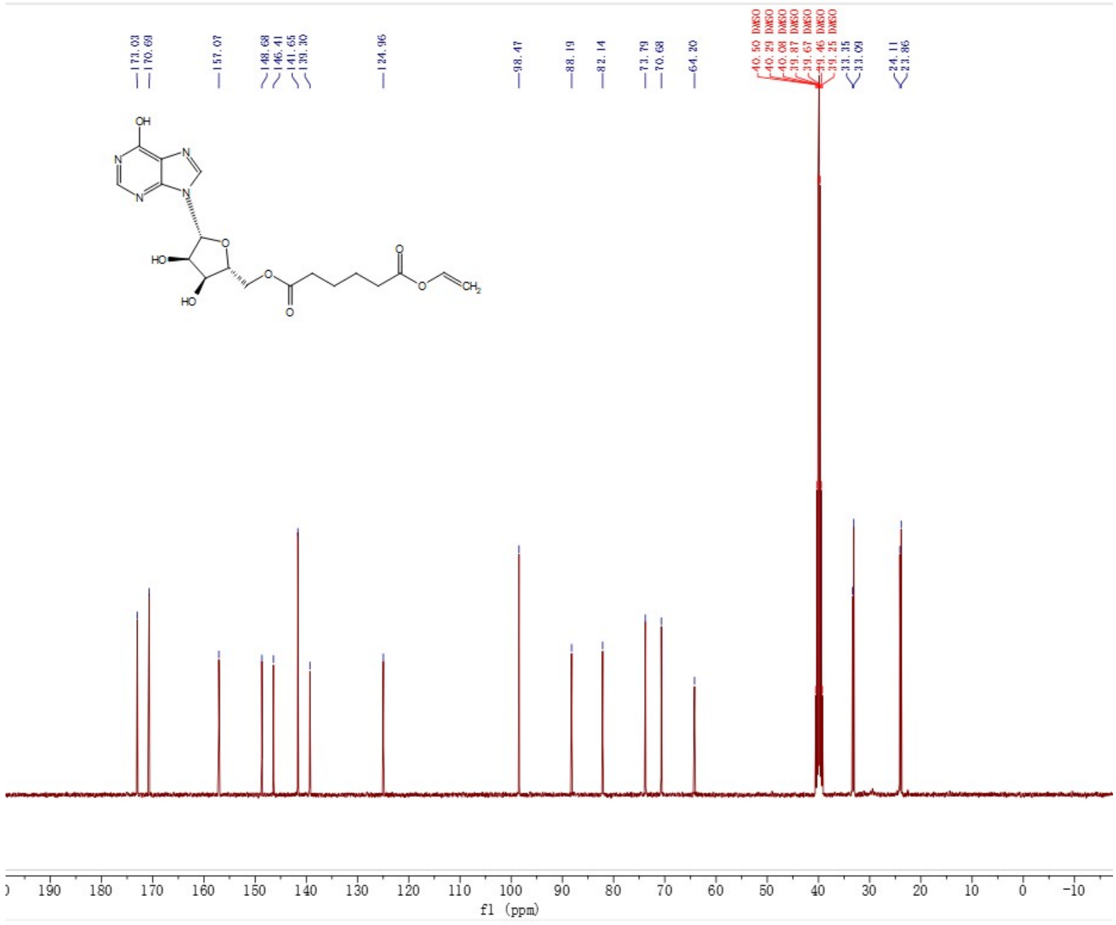

3i

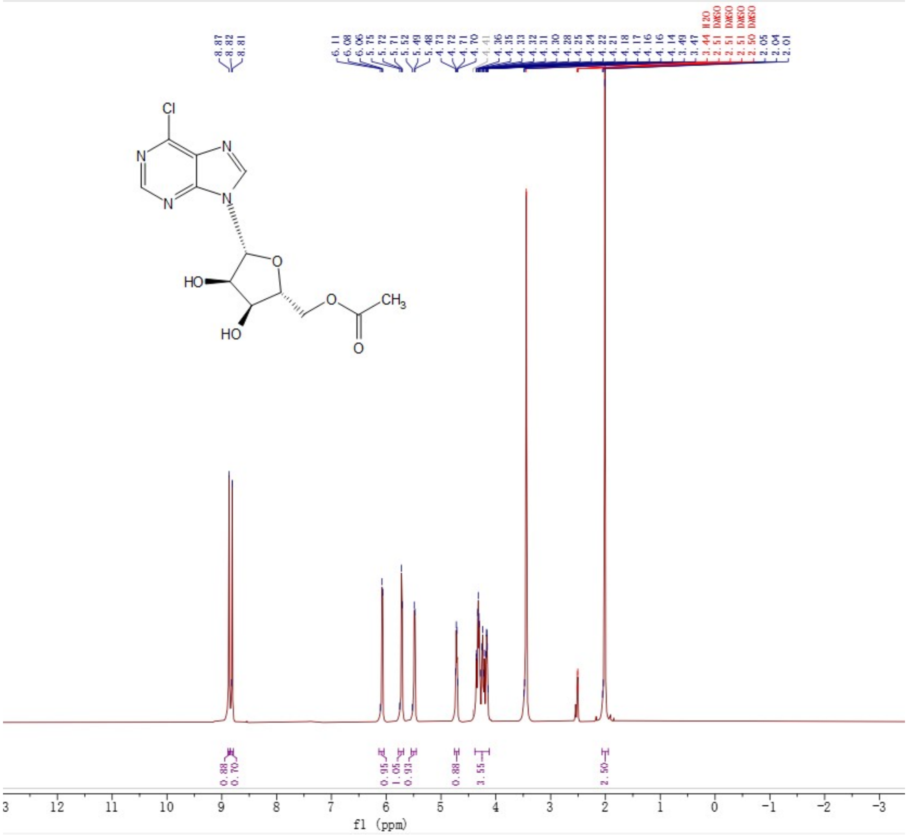
Cl
N
N
N
N
HO
HO
O
O
CH3
O
8.87
8.82
8.81
0.88
0.70
0.95
1.05
0.93
0.88
3.55
2.50
f1 (ppm)

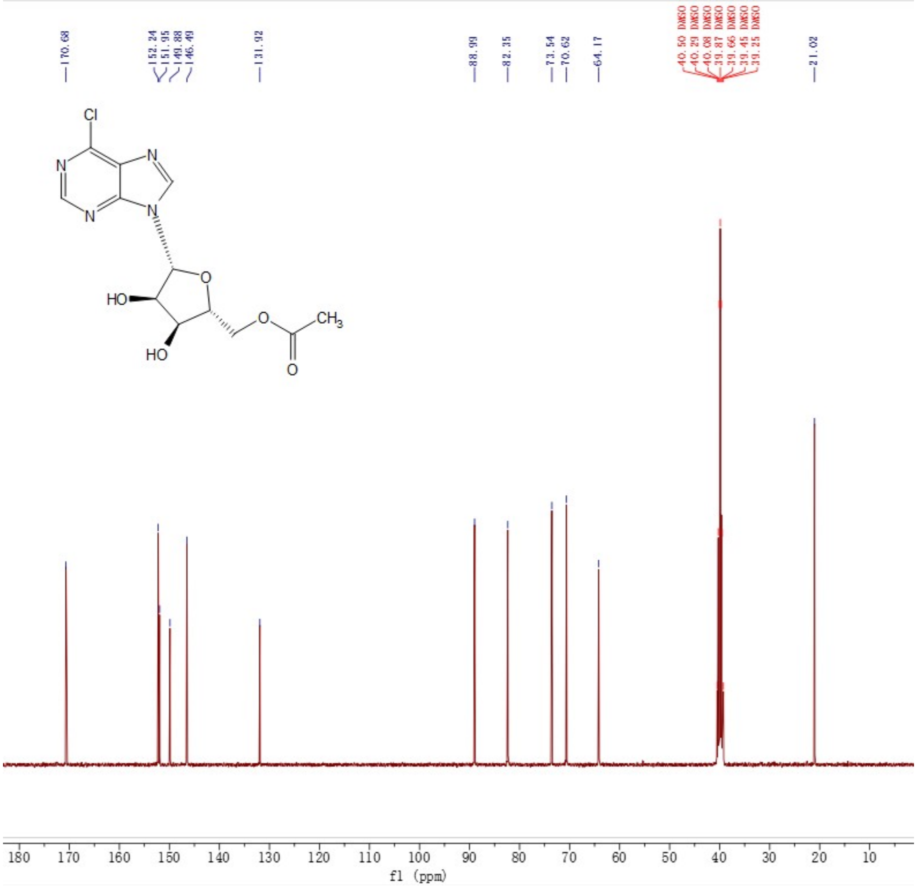
170.68
152.24
151.95
149.88
146.49
131.92
88.99
82.35
73.54
70.62
64.17
21.02
Cl
N
N
N
N
HO
HO
O
O
CH3
O
f1 (ppm)

3j

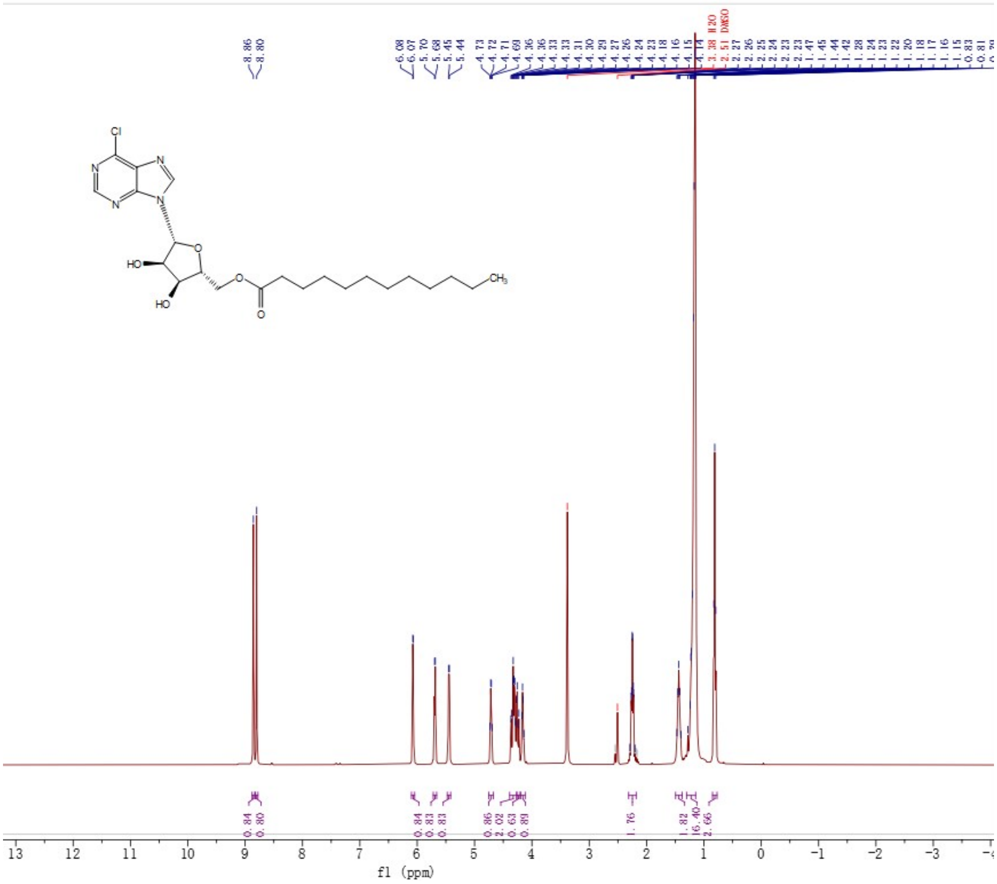

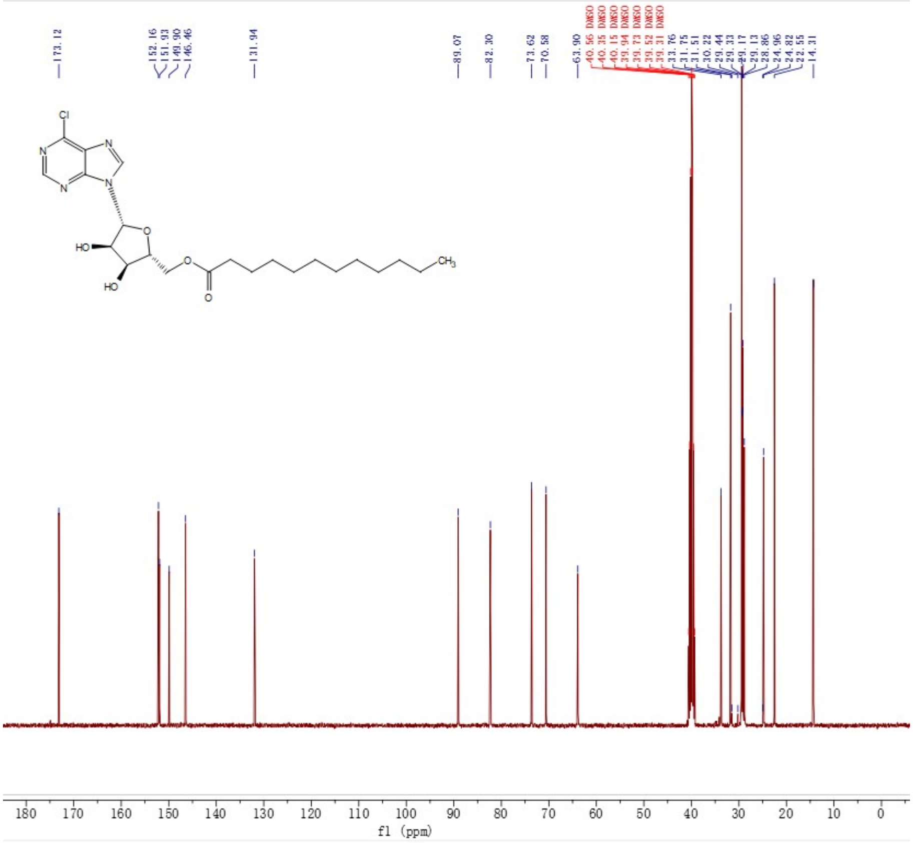

3k

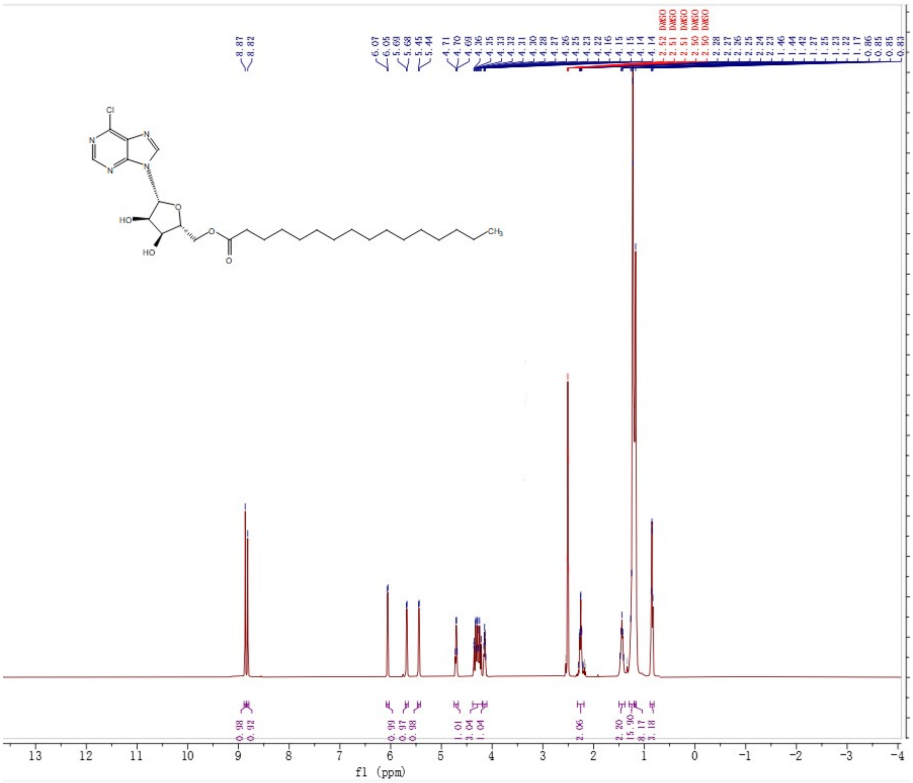
8.87
8.82
6.07
6.05
5.69
5.68
5.45
5.44
4.71
4.70
4.69
4.36
4.35
4.33
4.32
4.31
4.30
4.28
4.27
4.26
4.25
4.23
4.22
4.16
4.15
4.15
4.14
4.14
2.52 DMSO
2.51 DMSO
2.51 DMSO
2.50 DMSO
2.50 DMSO
2.28
2.27
2.26
2.25
2.24
2.23
1.46
1.44
1.42
1.27
1.25
1.23
1.22
1.17
0.86
0.85
0.83
Cl
N
HO
O
CH3
0.98
0.92
0.99
0.97
0.98
1.01
3.04
1.04
2.06
2.20
15.90
8.17
3.18
13 12 11 10 9 8 7 6 5 4 3 2 1 0 −1 −2 −3 −4
f1 (ppm)

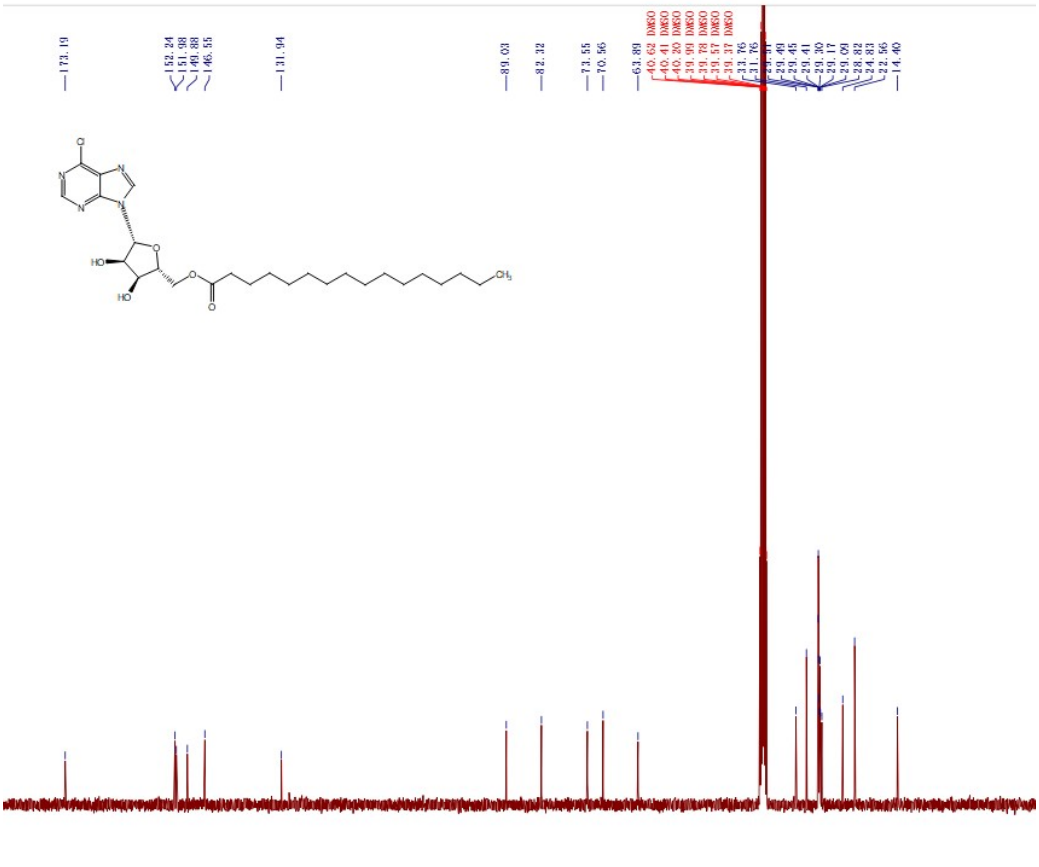
173.19
152.24
151.98
149.88
146.55
131.94
89.03
82.32
73.55
70.56
63.89
40.62 DMSO
40.41 DMSO
40.20 DMSO
39.99 DMSO
39.78 DMSO
39.57 DMSO
39.37 DMSO
33.76
31.76
29.49
29.45
29.41
29.30
29.17
29.09
28.82
24.83
22.56
14.40
Cl
N
HO
O
CH3
180 170 160 150 140 130 120 110 100 90 80 70 60 50 40 30 20 10 0 −10
f1 (ppm)

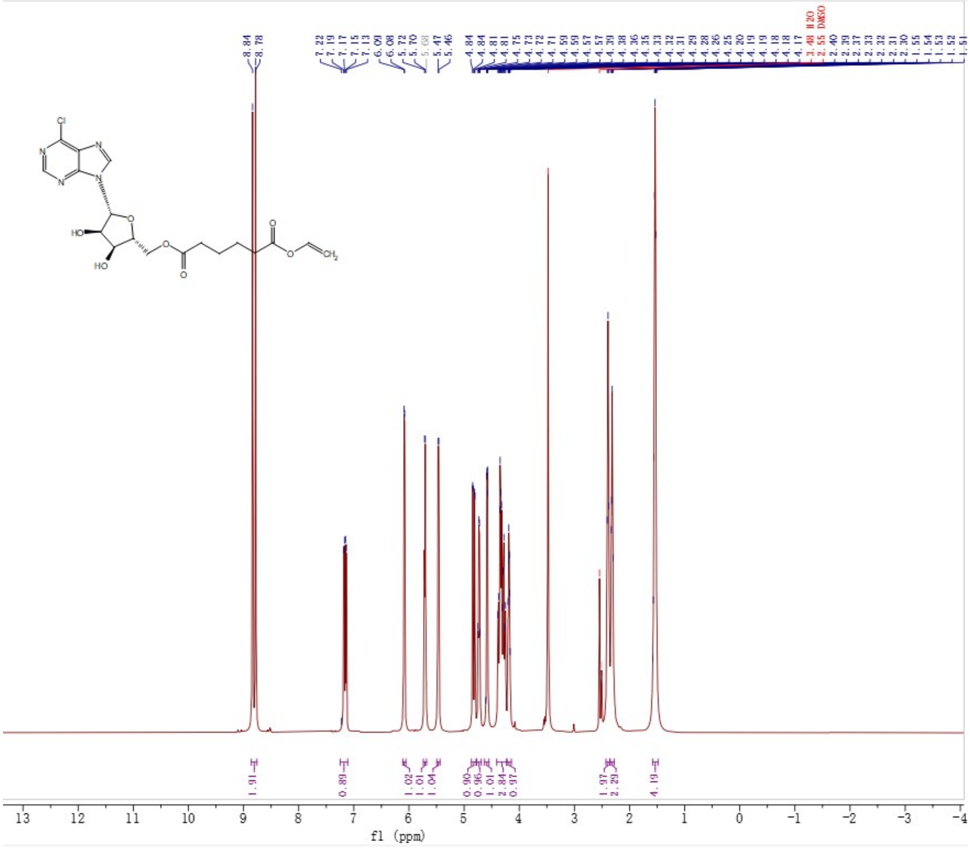
8.84
8.78
7.22
7.19
7.17
7.15
7.13
6.09
6.08
5.72
5.70
5.68
5.47
5.46
3.48 H2O
2.55 DMSO
1.91
0.89
1.02
1.01
1.04
0.90
0.96
1.01
2.84
0.97
1.97
2.29
4.19
f1 (ppm)

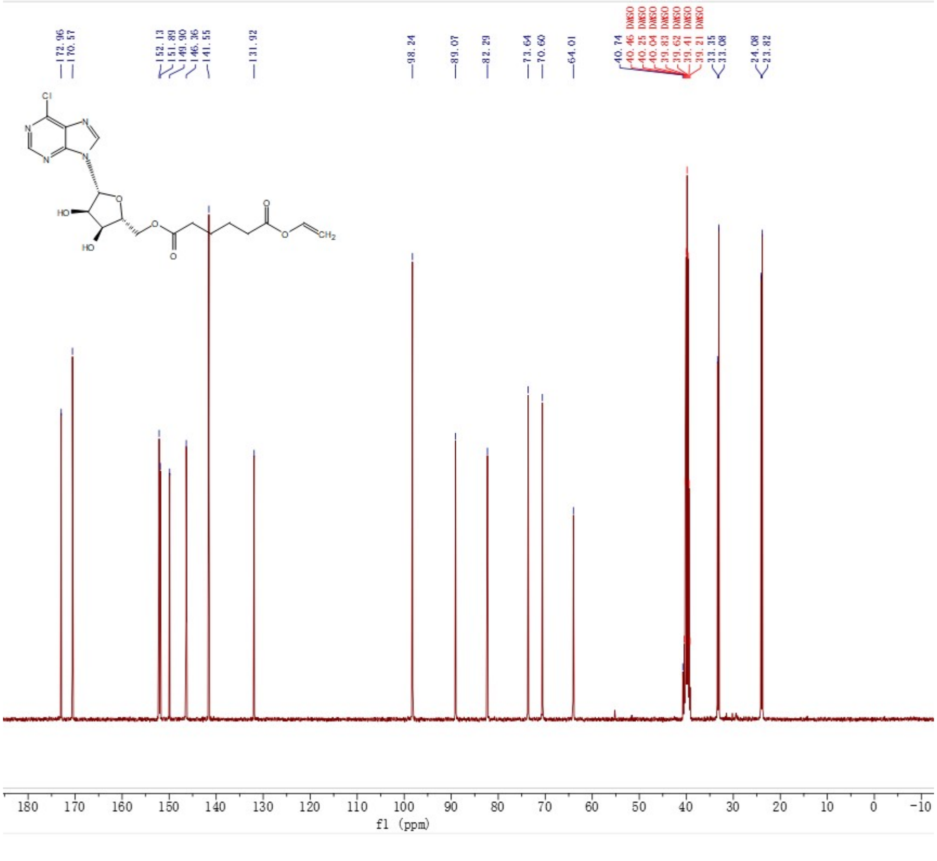
172.96
170.57
152.13
151.89
149.90
146.36
141.55
131.92
98.24
89.07
82.29
73.64
70.60
64.01
40.74
40.46 DMSO
40.25 DMSO
40.04 DMSO
39.83 DMSO
39.62 DMSO
39.41 DMSO
39.21 DMSO
33.35
33.08
24.08
23.82
f1 (ppm)
